# IL-1β-activated mTORC2 promotes accumulation of IFN-γ^+^ γδ T cells by upregulating CXCR3 to restrict hepatic fibrosis

**DOI:** 10.1038/s41419-022-04739-3

**Published:** 2022-04-01

**Authors:** Qihui Liu, Quanli Yang, Zengfeng Wu, Yanfang Chen, Miaomiao Xu, Hua Zhang, Jiliang Zhao, Zonghua Liu, Zerong Guan, Jing Luo, Zhi-yong Li, Guodong Sun, Qiong Wen, Yan Xu, Zhenhua Li, Kebing Chen, Xiaosong Ben, Wanchun He, Xueshi Li, Zhinan Yin, Jianlei Hao, Ligong Lu

**Affiliations:** 1 Zhuhai Institute of Translational Medicine, Zhuhai People’s Hospital Affiliated with Jinan University, Jinan University, Zhuhai, 519000 Guangdong China; 2 The Biomedical Translational Research Institute, Faculty of Medical Science, Jinan University, Guangzhou, 510632 Guangdong China; 3 Nanomedicine Translational Research Center, China-Japan Union Hospital of Jilin University, 126 Sendai Street, Changchun, 130033 Jilin China; 4 State Key Laboratory of Medicinal Chemical Biology, College of Life Sciences, Nankai University, Tianjin, 300071 China; 5 Department of Orthopedics, The Fifth Affiliated Hospital (Heyuan Shenhe People’s Hospital), Jinan University, Heyuan, 517000 China; 6 Department of Orthopedics, The First Affiliated Hospital, Jinan University, Guangzhou, 510632 Guangdong China; 7 Department of Spine Surgery, Center for Orthopaedic Surgery, The Sixth Affiliated Hospital of Sun Yat-sen University, Guangzhou, 510655 Guangdong China; 8 Department of Thoracic Surgery, Guangdong Provincial People’s Hospital, Guangdong Academy of Medical Sciences, Guangzhou, 510080 Guangdong China; 9 Guangdong Second Traditional Medicine Hospital, Guangzhou, 510095 Guangdong China; 10 Zhuhai Interventional Medical Center, Zhuhai Precision Medical Center, Zhuhai People’s Hospital, Zhuhai Hospital Affiliated with Jinan University, Zhuhai, Guangdong 519000 China

**Keywords:** Chemotaxis, Liver fibrosis

## Abstract

Liver fibrosis represents a severe stage of liver damage, with hallmarks of inflammation, hepatic stellate cell activation, and extracellular matrix accumulation. Although previous studies demonstrated γδ T cells are involved in liver fibrosis, the precise role and mechanisms of γδ T cells migrating to fibrotic liver have not been elucidated. Here, we aim to investigate the functional subsets of γδ T cells in hepatic fibrosis and to further explore the underlying causes and drivers of migration. In this study, we observed that γδ T cells accumulate in fibrotic liver. Adoptive transfer of γδ T, especially Vγ4 γδ T subset, can significantly alleviate liver fibrosis. In addition, CCl_4_ treatment also leads to activation of mTOR signaling in γδ T cells. Genetic deletion of the Rictor gene, but not Raptor, in γδ T cells markedly exacerbated liver fibrosis. Mechanistically, CCl_4_-induced liver injury causes macrophage accumulation in the liver, and IL-1β produced by macrophages promotes mTORC2 signaling activation in γδ T cells, which upregulates T-bet expression and eventually promotes CXCR3 transcription to drive γδ T cell migration. Moreover, hepatic γδ T cells ameliorated liver fibrosis by cytotoxicity against activated hepatic stellate cells in FasL-dependent manner, and secrete IFN-γ to inhibit the differentiation of pro-fibrotic Th17 cells. Thus, IL-1β-activated mTORC2 signaling in γδ T cells upregulates CXCR3 expression, which is critical for IFN-γ^+^ γδ T cells migration into the liver and amelioration of liver fibrosis. Our findings indicate that targeting the mTORC2 or CXCR3 in γδ T cells could be considered as a promising approach for γδ T cell immunotherapy against liver fibrosis.

## Introduction

Fibrosis occurs in almost every part of the body and causes organ failure in the context of most chronic diseases [1]. The damaged liver undergoes a wound healing process, with the accumulation of extracellular matrix and collagen, which leads to organ dysfunction [2]. Activation of hepatic stellate cells (aHSCs) is a major event for fibrosis formation [3]. Thus, studying the regulation of HSCs activation is an important potential therapeutic target for liver fibrosis.

Immune cells and immune responses are critically involved in liver fibrosis [2, 4]. During the liver fibrosis process, macrophages, neutrophils, NK cells and T cells are involved and contribute to the inflammation and activation of HSCs [5]. The heterogeneity of T cells, with complicated effector molecules, plays a key role in disease outcome [6]. Therefore, precisely studying the function of a specific T cell subset is essential to understanding the pathogenesis of liver fibrosis.

γδ T cells, although expressing TCRs, are characterized as innate immune cells and exert critical functions in diseases [7]. γδ T cells are divided into γδ T1 and γδ T17 functional subsets, which are defined by IFN-γ and IL-17 production, respectively. Our previous studies identified γδ T cells, especially Vγ4 subset, as important IFN-γ producers in tumor immunity, preferentially in the early stage [8]. It has been reported that γδ T cells are protective in liver fibrosis, through both direct inducing apoptosis of HSCs and enhancing NK cell-mediated cytolysis of HSCs [9]. However, the precise role of γδ T cells regarding the TCR-based subsets remains to be elucidated.

Mammalian/mechanistic target of rapamycin (mTOR) complexes are important orchestrators of the metabolism of immune cells, governing cell differentiation and function [10]. Previously, we found that mTORC1 supported γδ T1 differentiation through regulating glycolysis, whereas mTORC2 activated γδ T17 differentiation by inhibiting mitochondrial ROS production [11]. The recruitment of immune cells to the liver is important for the function of regulating liver fibrosis. It has been reported that mTOR signaling mediates the expression of chemokine receptors on immune cells [12]. However, the potential role of mTOR in γδ T cells during liver fibrosis is not well characterized.

Although it is known that γδ T cells produce IFN-γ to inhibit the liver fibrosis [13], it is not clear how the γδ T cells migrate to the fibrotic liver. CXCR3 is a receptor preferentially expressed on the surface of immune cells, such as monocytes, T cells, NK cells and dendritic cells. CXCL10, known as interferon γ-induced protein 10, is one of the selective ligands for CXCR3. The CXCR3/CXCL10 axis regulates immune cell migration, differentiation, and activation [14, 15]. However, whether CXCR3/CXCL10 axis regulates IFN-γ^+^ γδ T cells accumulation in fibrotic liver remains unknown.

In this study, we found that γδ T cell produced IFN-γ inhibited pro-fibrotic Th17 cells in liver fibrosis. And macrophage-derived IL-1β contributes to mTORC2 activation of γδ T cells, which is required for IFN-γ^+^ γδ T cells to migrate to the fibrotic liver through upregulating of CXCR3. Thus, we found previously unappreciated mechanisms of γδ T cells migrate into the liver and exert a protective effect against liver fibrosis. Targeting mTORC2 pathways may provide important clues for developing therapeutics to inhibit liver fibrosis.

## Materials and methods

### Animals

B6.Cg-Rptor^tm1.1Dmsa^/J (B6 Raptor-flox), Rictor^tm1.1Klg^/SjmJ (B6 Rictor-flox), B6.Cg-Tg (CD2-icre) 4Kio/J (hCD2-iCre), B6.129S7-Ifngtm1Ts/J (IFN-γ^-/-^), C57BL/6-Il17atm1Bcgen/J (IL-17A^-/-^), B6.129S4-Ifngtm3.1Lky/J (IFN-γ-eYFP), C57BL/6-Il17atm1Bcgen/J (IL-17-GFP), B6.129P2-Cxcr3tm1Dgen/J (CXCR3^-/-^), B6.129S6-Tbx21tm1Glm/J (Tbx21^-/-^) and B6.129P2-Tcrd^tm1Mom^/J (TCR δ^−/−^) mice were purchased from The Jackson Laboratory (Bar Harbor, ME). CD45.1 mice were given by Zhongjun Dong from Tsinghua University (Beijing, China). Sex- and age-matched animals were randomly assigned to different groups. All mice were maintained under SPF conditions and all animal procedures were approved by the Institutional Animal Care and Use Committee of Jinan University.

### Reagents

Recombinant mouse (rm)IL-2 (Peprotech), purified anti-mouse CD3 mAb (145-2C11), anti-mouse CD28 mAb (PV1), hamster anti-mouse TCR Vγ1 mAb (2.11), and hamster anti-mouse TCR Vγ4 mAb (UC3) were from Sungene Biotech (Tianjin, China). FITC-conjugated anti-mouse CD4 (RM4-5), and PE-conjugated anti-mouse γδ TCR (GL3), APC-conjugated anti-mouse NKG2D (CX5), eFlour450-conjugated anti-mouse CD3 (17A2), Percpcy5.5-conjugated anti-mouse IL-17 mAb (eBio17B7) and Foxp3 staining buffer set were purchased from eBioscience (San Diego, CA, USA). APC/Percpcy5.5-conjugated anti-mouse CD4 (GK1.5), FITC/APC-conjugated anti-mouse TCR γδ mAb (GL3), PE-Cy7-conjugated anti-mouse IFN-γ mAb (XMG1.2), PE-Cy7-conjugated anti-mouse CD3 mAb (145-2C11), APC-conjugated anti-mouse Vγ4 mAb (UC3-10A6), PE anti-mouse CXCR3 mAb (CXCR3-173), Percpcy5.5 anti-mouse CCR3 mAb (J073E5), Alexa Fluor 647 anti-mouse CCR5 mAb (HM-CCR5), Brilliant Violet 510 anti-mouse CCR2 mAb (SA203G11), PE-Cy7 anti-mouse CXCR5 (L138D7) and PE-Cy7 anti-mouse CXCR6 (SA051D1), Alexa Fluor 647 anti-mouse F4/80 mAb (BM8), and PE anti-mouse Ly6G mAb (1A8) were purchased from Biolegend (San Diego, CA, USA). PE-conjugated anti-mouse p-Akt473 mAb (D9E), Alexa Fluor 647- conjugated anti-mouse p-Akt473 mAb (D9E) and Alexa Fluor 488-conjugated anti-human/mouse p-S6 mAb (D57.2.2E) were purchased from Cell Signaling Technology (Danvers, MA, USA). PMA/ionomycin were purchased from Sigma, Inc. (St. Louis, MO, USA), GolgiStop was purchased from BD Biosciences (San Jose, CA, USA), Anti-alpha smooth muscle Actin antibody was purchased from Abcam (Cambridge, UK).

### Induction of acute liver injury and fibrosis

For induction of liver fibrosis, 6–8-week-old male mice were injected intraperitoneally (i.p.) with CCl_4_ (0.6 mL/kg body weight, dissolved in corn oil at a ratio of 1:9) (Aladdin, Shanghai, China) or vehicle (corn oil) three times a week for 4 weeks. For toxic acute liver injury, mice were given i.p. injection of a double dose of CCl_4_, and the mice were euthanized 48 h after CCl_4_ injection. For genetic mouse experiments, age- and sex-matched WT and TCRδ^−/−^ mice were co-housed at a 1:1 ratio for at least 4 weeks to minimize the potential microbiome effects. The sample size was chosen to ensure the possibility of statistical analysis and to also minimize the use of animals in accordance with the animal experiment committee of Jinan University. The results from previous results were also used to determine the sample size.

### Isolation of liver nonparenchymal cells

Isolation of liver nonparenchymal cells (NPCs) was performed following an established method [16]. In brief, mice were anesthetized, the inferior vena cava was clamped, and a 20-G catheter was inserted into the superior vena cava. The liver was subsequently perfused with 1× Hank’s balanced salt solution (HBSS) followed by a digestion buffer (1× HBSS supplemented with 0.05% collagenase [Type IV; Sigma], 30 U/mL DNase (Sigma), 1.25 mM CaCl_2_, 4 mM MgSO_4_, and 10 mM 4-[2-hydroxyethyl]-1-piperazine ethane sulfonic acid). The liver was cut into 2 mm^3^ pieces and shaken at 100 rpm for 30 min in a 37 °C incubator. Single-cell suspensions were filtered through a 70-µm cell strainer, then fractionated with 30% Percoll (Sigma) at 1.04 g/mL, NPCs were resuspended in erythrocyte lysis buffer, and washed with HBSS. The NPCs suspension was used for flow cytometric analysis.

### Statistical analysis

All quantitative data were shown as mean ± SD unless otherwise indicated. All samples were compared using two-tailed, unpaired Student’s *t*-test between two groups or one-way ANOVA for comparison of two or multiple groups, respectively. The following terminology is used to show statistical significance: *P < 0.05, **P < 0.01, ***P < 0.001. No samples or animals were excluded from the analysis. Statistical analysis was performed with GraphPad Prism software. Other detailed materials and methods are described in the Supplementary information.

## Results

### Vγ4 cells protected mice from liver fibrosis through IFN-γ production and FasL-mediated cytotoxicity

**Fig. 1:**
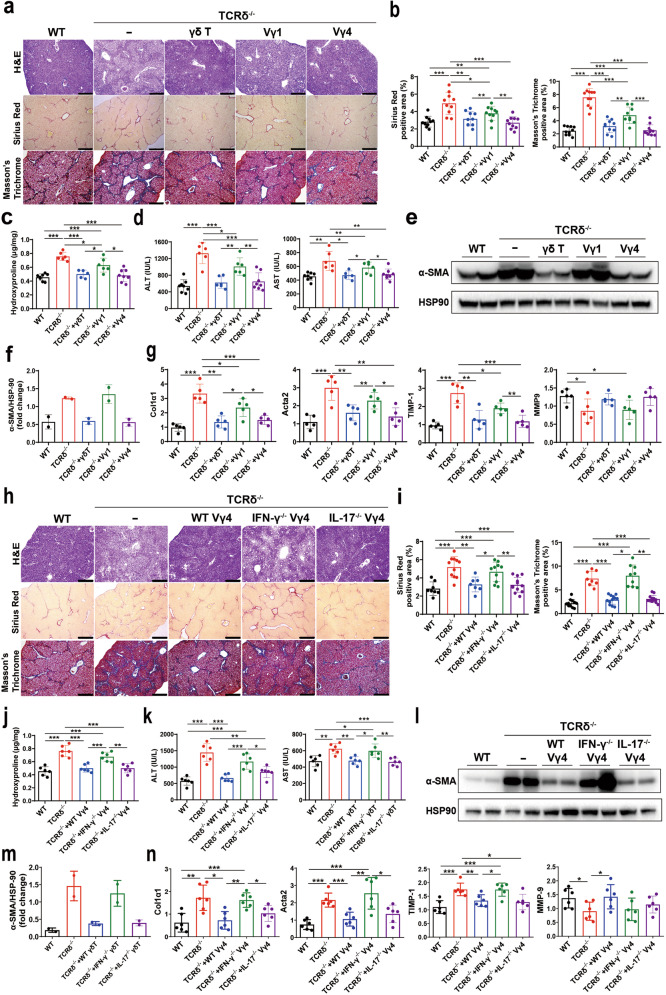
IFN-γ^+^ Vγ4 cells play a protective role in CCl_4_-induced liver fibrosis. **a**–**g** Wild-type (WT), TCRδ^-/-^ or TCRδ^-/-^ mice reconstituted with 5 × 10^5^ γδ T cells or Vγ1 or Vγ4 cells, and repetitive CCl_4_ were challenged twice weekly for 4 weeks (*n* = 5–8/group; 3 replicates). **a** Representative liver histology of H&E, Sirius Red staining and Masson’s Trichrome staining (bar = 500 μm). **b** Sirius Red staining and Masson’s Trichrome staining were quantified by ImageJ (National Institutes of Health, MD, USA) analysis, counted in ten different fields for each sample, two samples from each mouse, and presented as fold change compared with the control. **c** Hydroxyproline content in liver tissues. **d** Serum ALT and AST levels. **e**, **f** Representative western bolt images and quantitative analysis of α-SMA expression in liver tissues. **g** qRT-PCR analysis of the relative expression of Col1α1, Acta2, TIMP-1 and MMP-9 in liver tissues. **h**–**n** WT, TCRδ^−/−^ or TCRδ^−/−^ mice reconstituted with 5 × 10^5^ WT Vγ4 or IFN-γ^-/-^ Vγ4 or IL-17^-/-^ Vγ4 cells, and repetitive CCl_4_ were challenged twice weekly for 4 weeks (*n* = 6/group; 3 replicates). **h** Representative liver histology of H&E, Sirius Red staining and Masson’s Trichrome staining (bar = 500 μm). **i** Sirius Red staining and Masson’s Trichrome staining were quantified by ImageJ. **j** Hydroxyproline content in liver tissues. **k** Serum ALT and AST levels. **l**, **m** Representative western bolt images and quantitative analysis of α-SMA expression in liver tissues. **n** qRT-PCR analysis of the relative expression of Col1α1, Acta2, TIMP-1 and MMP-9 in mouse liver. H&E hematoxylin and eosin, ALT alanine aminotransferase, CCl_4_ carbon tetrachloride, qRT-PCR quantitative reverse-transcription PCR, WT wild-type. Data are presented as the mean ± SD. *P < 0.05, **P < 0.01, and ***P < 0.001 in comparison with the corresponding controls, by unpaired Student’s *t*-test between two groups or one-way ANOVA for comparison of two or multiple groups, respectively.

To define the role of γδ T cells in chronic liver disease, we first investigated fibrogenesis in γδ T cell-deficient mice (TCRδ^-/-^), by repetitive CCl_4_ treatment. WT mice showed more inflammatory responses in the liver (Supplementary Fig. S1a–e), but there were no differences in the apoptosis and proliferation of γδ T cells after Oil or CCl_4_ administration (Supplementary Fig. S1f, g). TCRδ^-/-^ mice showed significantly increased infiltration of inflammatory cells and fibrosis formation, and the disease progression was restored by adoptive transfer of γδ T cells upon CCl_4_ administration, but there were no differences between the WT and TCRδ^-/-^ mice in the extent of fibrosis in the untreated liver (Supplementary Fig. S1h–n), which suggests that γδ T cells are sufficient to protect mice from liver fibrosis. Next, we firstly compared the function of Vγ1 and Vγ4 in protecting liver fibrosis. When Vγ1 or Vγ4 cells were transferred respectively to TCRδ^-/-^ mice, both liver fibrosis were ameliorated, whereas the Vγ4 subset showed slightly stronger, as assessed by H&E, Sirius Red and Masson’s Trichrome staining, compared with Vγ1 reconstituted mice (Fig. 1a, b). In addition, there was a significant reduction in hydroxyproline, serum ALT and AST levels, hepatic protein level of α-SMA and mRNA levels of Col1α1, Acta2, TIMP-1 in Vγ4 cells reconstituted mice, while Vγ1 reconstitution also had a slight therapeutic effect (Fig. 1c–g), which indicating TCR-based specific subset of γδ T cells is playing a unique function in liver fibrosis.

Next, we sought to demonstrate the effector molecules of γδ T cells in this process. IFN-γ and IL-17 are two well-established effector molecules produced by γδ T cells [17], both cytokines were found to have an opposing function in liver fibrosis: IFN-γ has anti-fibrotic properties, while IL-17 has pro-fibrotic properties. Vγ4 cells, which have a stronger anti-fibrotic effect, were sorted from WT, IFN-γ^-/-^ and IL-17^-/-^ mice, expanded in vitro and transferred into TCRδ^-/-^ mice, and these chimeric mice were induced liver fibrosis. The result showed that IFN-γ^-/-^ Vγ4 cells completely lost anti-fibrotic function compared with WT Vγ4 cells and IL-17^-/-^ Vγ4 cells reconstituted mice, as assessed by H&E, Sirius Red and Masson’s Trichrome staining (Fig. 1h, i). Consistently, the upregulation of hydroxyproline, serum ALT and AST levels, and hepatic protein level of α-SMA and mRNA levels of Col1α1, Acta2 and TIMP-1 were also increased in IFN-γ^-/-^ Vγ4 cells reconstituted mice (Fig. 1j–n), while IL-17^-/-^ γδ T cells still showed protection against hepatic fibrosis (Fig. 1h–n). These results indicate that IFN-γ producing γδ T cells, preferentially produced by the Vγ4 subset, plays an important protective role in CCl_4_-induced liver fibrosis.

To further study the protective mechanisms of γδ T cells during liver fibrosis. We investigated the interaction between γδ T cells and aHSCs by coculture γδ T with JS1 cells, a murine aHSC cell line, for 48 h. Consistent with the in vivo function of Vγ1 and Vγ4 cells (Fig. 1a), both Vγ1 and Vγ4 are able to induce aHSCs apoptosis after cell-to-cell contact, and the Vγ4 subset showed slightly stronger cytolysis ability (Supplementary Fig. S2a, b). The previous publication showed that FasL was essential for γδ T cell-mediated cytotoxicity against aHSCs [13], we also found CCl_4_ treatment improved FasL expression in γδ T cells (Supplementary Fig. S2c, d), and blocking FasL in γδ T cells partially reduced cytotoxicity against aHSCs (Supplementary Fig. S2e), indicating a role for FasL expression on γδ T cells in inducing aHSCs apoptosis in vitro. Interestingly, both Vγ1 and Vγ4 cells lyse aHSCs via FasL, but not via NKG2D (Supplementary Fig. S2e), which is different from that the interactions between γδ T cells and tumor as we previously reported [18]. We also found that IFN-γ may be dispensable for γδ T cell-mediated cytotoxicity against aHSCs in vitro (Supplementary Fig. S2e). However, whether IFN-γ affects the activation and proliferation of HSCs in vivo still needs to be further explored. Thus, γδ T cells inhibit the activation of HSC by secreting IFN-γ and induce apoptosis of aHSCs by upregulating FasL expression to ameliorate liver fibrosis.

### CXCR3-mediated IFN-γ^+^ γδ T cells accumulate in chronic liver injury

**Fig. 2:**
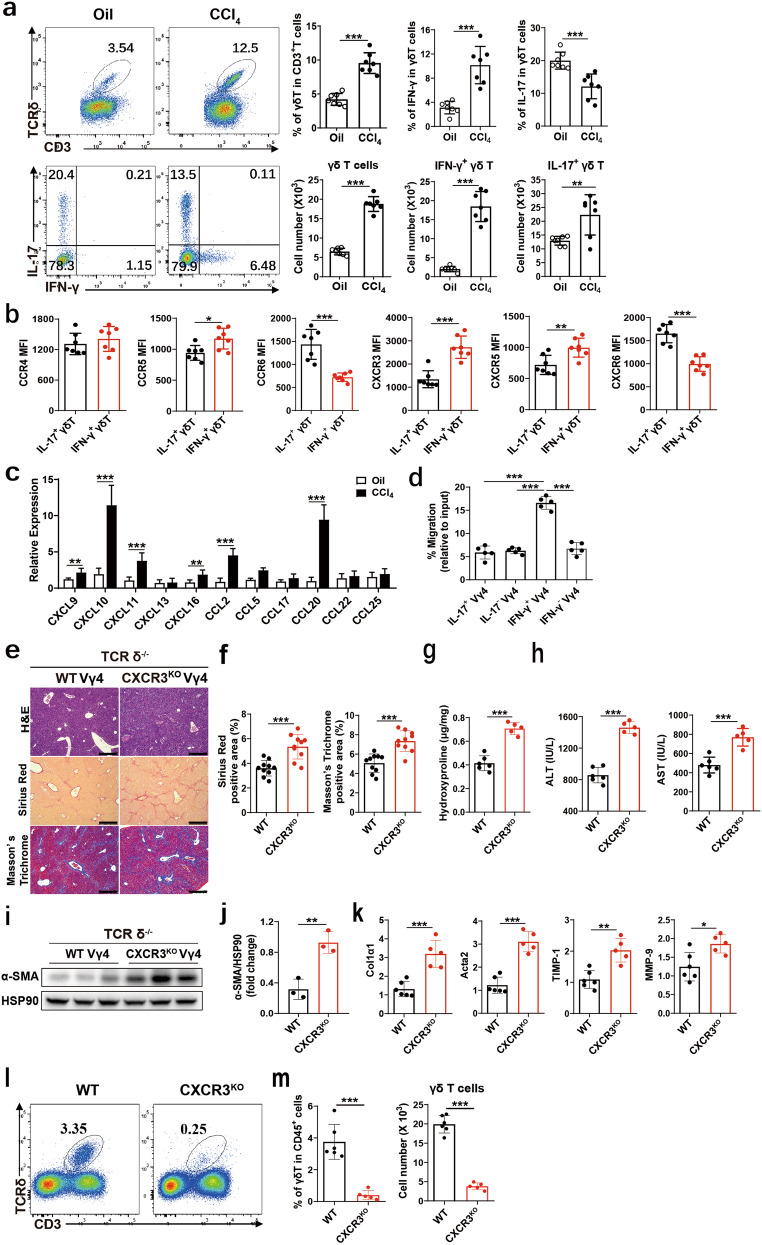
CXCR3-mediated IFN-γ^+^ γδ T cells accumulated in chronic liver injury. **a**–**c** WT mice were treated with CCl_4_ twice weekly for 4 weeks (*n* = 7/group; 3 replicates). **a** Representative FACS plots of γδ T cells, IFN-γ and IL-17A in γδ T cells in the liver of WT mice treated with Oil or CCl_4_. CD45^+^ CD3e^+^ TCR δ^+^ cells were gated. Statistical analysis of the percentage and the absolute cell number of γδ T cells in CD3^+^ leukocytes, IFN-γ and IL-17A in γδ T cells in liver tissue. **b** The mean fluorescence intensity (MFI) was determined by flow cytometry showing the expression of CCR2, CCR5, CCR6, CXCR3, CXCR5, and CXCR6 on IFN-γ^+^ γδ T and IL-17^+^ γδ T cell subsets in liver form CCl_4_-treated mice. **c** qRT-PCR analysis of the relative expression of chemokine genes in mouse liver form Oil or CCl_4_-treated mice. **d** IFN-γ^+^ Vγ4, IFN-γ Vγ4, IL-17^+^ Vγ4 and IL-17^-^ Vγ4 cells were sorted from IFN-γ-eYPF and IL-17-GPF mice, respectively, and those cells in response to CXCL10 (100 ng/mL) were assessed in transwell chambers for 3 h. **e**–**m** TCRδ^-/-^ mice were reconstituted with 5 × 10^5^ WT Vγ4 or CXCR3^KO^ Vγ4 cells, and repetitive CCl_4_ were challenged twice weekly for 4 weeks (*n* = 5–6/group; 3 replicates). **e** Representative liver histology of H&E, Sirius Red staining and Masson’s Trichrome staining (bar = 500 μm). **f** Sirius Red staining and Masson’s Trichrome staining were quantified by ImageJ. **g** Hydroxyproline content in liver tissues. **h** Serum ALT and AST levels. **i**, **j** Representative western bolt images and quantitative analysis of α-SMA expression in liver tissues. **k** qRT-PCR analysis of the relative expression of Col1α1, Acta2, TIMP-1 and MMP-9 in mouse liver. **l** Representative FACS plots of γδ T cells in the liver of TCRδ^-/-^ mice reconstituted with WT and CXCR3^KO^ Vγ4 cells. CD45^+^ CD3e^+^ TCRδ^+^ cells were gated. **m** Statistical analysis of the percentage and absolute cell number of γδ T cells in CD45^+^ leukocytes in liver tissue. Data are shown as mean ± SD. *P < 0.05, **P < 0.01, and ***P < 0.001 in comparison with the corresponding controls, by unpaired Student’s *t*-test between two groups.

Since the migration of γδ T cells into the fibrotic liver was shown significantly increased, the absolute numbers of IFN-γ^+^ γδ T and IL-17^+^ γδ T were increased 6- and 1.7-fold respectively, compared with those of Oil-treated mice (Fig. 2a), and this process is not dependent on the proliferation and apoptosis of hepatic γδ T cells (Supplementary Fig. S1f, g). Therefore, enhanced chemotaxis could be the reason for the increased IFN-γ^+^ γδ T cells in the fibrotic liver. We initially compared the profiles of chemokine receptors expression between IFN-γ^+^ γδ T and IL-17^+^ γδ T cells, and found that CCR5, CXCR3 and CXCR5 are highly expressing chemokine receptors in IFN-γ^+^ γδ T cells, especially, CXCR3 is highly expressed in IFN-γ^+^ γδ T cells, while CCR6 and CXCR6 are highly expressing chemokine receptors in IL-17^+^ γδ T cells (Fig. 2b). Next, we assessed gene expression of the corresponding ligands of the chemokine receptors, CXCL9, CXCL10, CXCL11, CXCL16, CCL2 and CCL20 are significantly upregulated, among which CXCL10 (the key ligand of CXCR3) and CCL20 (the ligand of CCR6) significantly increased in fibrotic liver tissue (Fig. 2c). Furthermore, we investigated whether the CXCL10-CXCR3 is necessary for IFN-γ^+^ γδ T cell migration, transwell assays were performed. In vitro expanded IFN-γ^+^ Vγ4, IFN-γ^-^ Vγ4, IL-17^+^ Vγ4 and IL-17^-^ Vγ4 cells were sorted from IFN-γ-eYPF and IL-17-GPF report mice, respectively, these cells migrated toward CXCL10 in transwell migration assays, the vast majority of Vγ4 cells that migrated in response to CXCL10 were IFN-γ^+^ Vγ4 cells but not IFN-γ^-^ Vγ4, IL-17^+^ Vγ4 and IL-17^-^ Vγ4 cells (Fig. 2d), suggesting that IFN-γ^+^ Vγ4 cells were preferentially mobilized by the coordinated action of CXCL10. To assess the role of CXCR3 expression on IFN-γ^+^ Vγ4 in vivo, IFN-γ^+^ γδ T cells from WT and CXCR3^-/-^ mice were transferred into TCRδ^-/-^ mice respectively, and liver fibrosis was induced. The result showed that Vγ4 cells with CXCR3 deficiency reconstituted mice (CXCR3^KO^) displayed significantly enhanced liver fibrosis, as assessed by H&E, Sirius Red and Masson’s Trichrome staining (Fig. 2e, f). Consistently, the upregulation of hydroxyproline, ALT and AST levels, and hepatic protein level of α-SMA and mRNA levels of Col1α1, Acta2 and TIMP-1 were also increased in CXCR3^KO^ mice (Fig. 2g–k). Furthermore, we found that CXCR3 deficiency significantly reduced the accumulation of IFN-γ^+^ γδ T cells in the fibrotic liver (Fig. 2l–m). Taken together, the results suggested that CXCR3 mediated the accumulation of IFN-γ^+^ γδ T into the fibrotic liver, which is important for the subsequent anti-fibrotic function of IFN-γ^+^ Vγ4 cells.

### mTORC2-mediated signaling is critical for γδ T cell migration into the injured liver by increasing CXCR3 expression

**Fig. 3:**
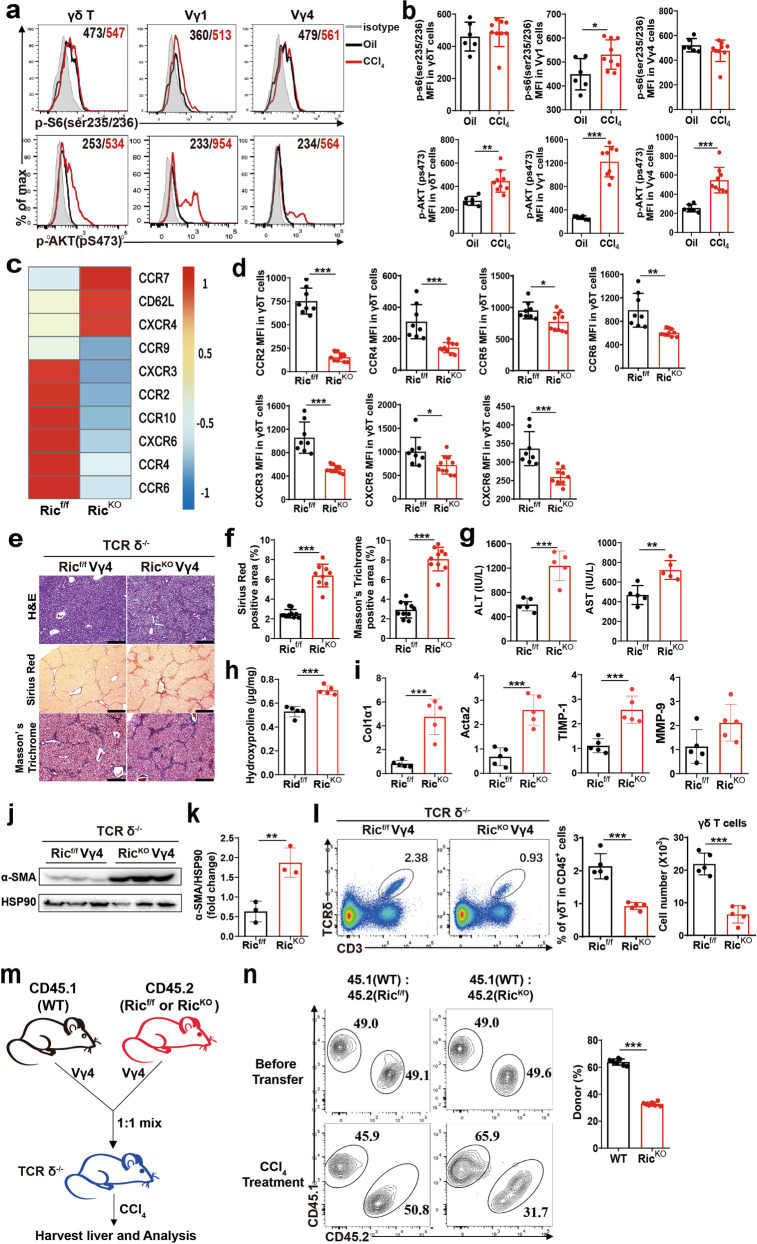
mTORC2-mediated signaling is critical for the migration of γδ T cells into the injured liver by increasing chemokine receptor expression. **a** Representative histograms and **b** MFI of p-S6 (ser235/236) and p-AKT (pS473) on γδ T, Vγ1 and Vγ4 cell subsets in the liver form Oil or CCl_4_-treated mice. CD45^+^ CD3e^+^ TCRδ^+^ cells were gated. **c** RNAseq heatmap of differentially expressed genes in samples of Ric^f/f^ (*n* = 6 mice per sample), Ric^KO^ (*n* = 5 mice per sample) γδ T cells isolated from pooled spleens. Expression of chemokine receptor genes for γδ T cells was shown. **d** The MFI was determined by flow cytometry showing the expression of CCR2, CCR4, CCR5, CCR6, CXCR3, CXCR5 and CXCR6 molecule expression on γδ T cells from Ric^f/f^ and Ric^KO^ mice. **e**–**i** TCRδ^-/-^ mice were reconstituted with Ric^f/f^ Vγ4 or Ric^KO^ Vγ4 cells, and repetitive CCl_4_ were challenged twice weekly for 4 weeks. **e** Representative liver histology of H&E, Sirius Red staining and Masson’s Trichrome staining. **f** Sirius Red staining and Masson’s Trichrome staining were quantified by ImageJ. **g** Serum ALT and AST levels. **h** Hydroxyproline content in liver tissues. **i** qRT-PCR analysis of the relative expression of Col1α1, Acta2, TIMP-1 and MMP-9 in mouse liver. **j**, **k** Representative western bolt images and quantitative analysis of α-SMA expression in liver tissues. **l** Representative FACS plots, statistical analysis of the percentage and the absolute cell number of γδ T cells in the liver. **m** Experimental scheme. Vγ4 cells from CD45.1(WT) and CD45.2 mice (Ric^f/f^ or Ric^KO^) were isolated and mixed at a ratio of 1:1 and injected (i.v.) into TCR δ^-/-^ hosts, and then host mice were injected (i.p) with a single dose of CCl_4_ for 24 h to induce liver injury. **n** Representative FACS plots of CD45.1^+^ Vγ4 and CD45.2^+^ Vγ4 cells were shown, and the percentages of donor-derived Vγ4 cells in the liver of the recipient mice were calculated. Data are shown as mean ± SD. *P < 0.05, **P < 0.01, and ***P < 0.001 in comparison with the corresponding controls, by unpaired Student’s *t*-test between two groups.

The mTOR cascade in immune cells involves migration and infiltration [12], and we have previously demonstrated that mTORC1 is a positive regulator of IFN-γ production by γδ T cells [11]. We then asked whether mTOR signaling regulates γδ T cell function in CCl_4_-induced liver fibrosis. To assess mTOR kinase activity within the fibrotic liver, CCl_4_ was injected into WT mice, and mTORC1 and mTORC2 activity in hepatic γδ T cells was determined by staining p-S6(235/236) and p-AKT(pS473), respectively. Interestingly, p-S6(ser235/236) was slightly activated in hepatic Vγ1 cells, and p-AKT(pS473) was conspicuously activated in hepatic Vγ1 and Vγ4 cells (Fig. 3a, b). The MFI (mean fluorescence intensity) of p-AKT(pS473) was increased approximately two-fold in both Vγ1 and Vγ4 subsets in the fibrotic liver, compared with those of Oil-treated mice (Fig. 3a, b). Consistently, mTORC2 but not mTORC1 is also activated in peripheral γδ T cell after CCl_4_ treatment (Supplementary Fig. S3).

To determine whether mTORC2 signaling is involved in the regulation of γδ T cell migration, γδ T cells from Rictor-f/f (Ric^f/f^) and Rictor-f/f-CD2-Cre (Ric^KO^) mice were sorted for RNAseq analysis. γδ T cells from Ric^KO^ showed that the expression of several chemokine receptors reduced, including CCR9, CXCR3, CCR2, CCR10, CXCR6, CCR4 and CCR6 (Fig. 3c), and the results were further validated by analyzing chemokine receptors expression on splenic γδ T cells isolated from Ric^f/f^ and Ric^KO^ mice (Fig. 3d), the MFI of CCR2, CCR4, CCR5, CCR6, CXCR3, CXCR5 and CXCR6 were reduced in Ric^KO^ γδ T cells. To further illustrate the mechanism of CXCR3 expression in γδ T cells, we next detected T-bet expression, which is required for CXCR3 expression in CD4^+^T cells [19] and γδ T cells (Supplementary Fig. S4a, b), in γδ T cells from WT and Ric^KO^ mice. The result showed that T-bet expression was significantly reduced in Ric^KO^ γδ T cells (Supplementary Fig. S5a, b). These analyses hinted at CXCR3-mediated γδ T cells accumulation is largely dependent on mTORC2 activity in chronic liver injury.

To further elucidate the potential role of mTORC1 and mTORC2 in regulating γδ T cells function, we reconstituted Raptor-f/f-CD2-cre (Rap^KO^) and Rictor-f/f-CD2-cre (Ric^KO^) Vγ4 cells into TCRδ^-/-^ mice respectively and then induced liver fibrosis. The results showed that Vγ4 cells with Raptor deficiency reconstituted mice had no significant effect on the progression of liver fibrosis compared with Rap^f/f^ Vγ4 cells reconstituted mice (Supplementary Fig. S6a–g). Strikingly, Vγ4 cells with Rictor deficiency reconstituted mice significantly aggravated the liver injury and fibrosis (Fig. 3e, f) as determined by H&E, Sirius Red and Masson’s Trichrome staining, compared with Ric^f/f^ Vγ4 cells reconstituted mice. Consistently, strongly upregulation of hydroxyproline level, ALT and AST activities in serum (Fig. 3g, h), hepatic protein level of α-SMA and mRNA level of Col1α1, Acta2 and TIMP-1 (Fig. 3i–k) was markedly increased in Ric^KO^ Vγ4 cells reconstituted mice.

Next, we investigated the mechanism by which mTORC2 signaling regulates γδ T cells to restrict liver fibrosis. The percentage and absolute number of hepatic γδ T cells were significantly reduced in Ric^KO^ Vγ4 cells reconstituted mice (Fig. 3l). We also investigated neutrophils, infiltrating macrophages and Th17 cells, which were significantly increased in Ric^KO^ Vγ4 cells reconstituted mice (Supplementary Fig. S7a, c), but the CD4^+^ T, CD8^+^ T, NK and NKT showed no differences (Supplementary Fig. S7b). However, In Ric^KO^ Vγ4 cells reconstituted mice, as anticipated, we observed no significant differences in γδ T cells, neutrophils, IM, CD4^+^T, CD8^+^T, NK and NKT cells, compared with Ric^f/f^ Vγ4 cells reconstituted mice (Supplementary Fig. S6h, i). Furthermore, we performed competitive adoptive transfer experiments and the result showed that cell-intrinsic mechanisms of mTORC2 in mediating the accumulation of Vγ4 cells in the injured liver (Fig. 3m, n). Our results indicate mTORC2 is required for γδ T cells accumulation and exerting protective effects in CCl_4_-induced liver fibrosis.

### IL-1β is important for γδ T cells mTORC2 activation and mobility

**Fig. 4:**
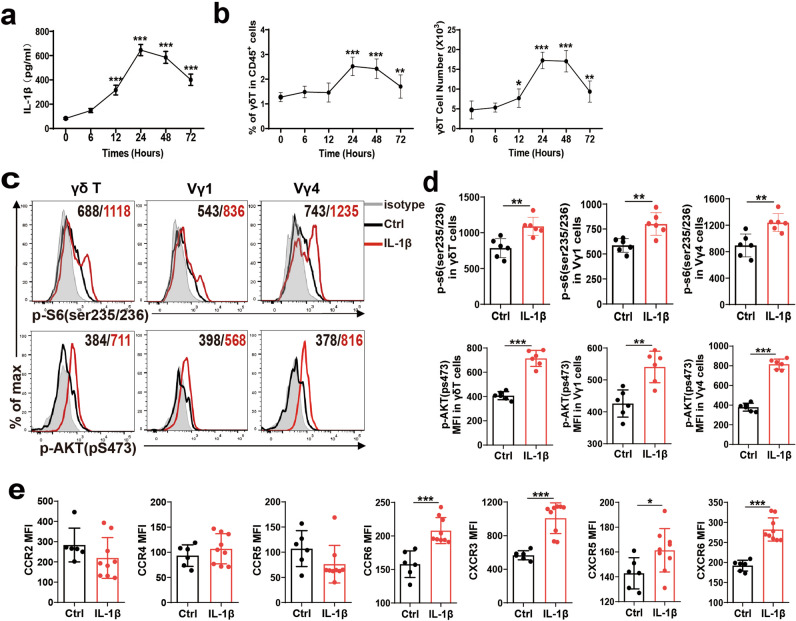
IL-1β-induced γδ T cell mTOR-signaling activation and chemokine receptor expression. **a** Serum was collected at the indicated time points after CCl_4_ treatment (*n* = 5/group; 3 replicates), and serum levels of IL-1β were determined by using enzyme-linked immunosorbent assay (ELISA) kits according to manufacturer’s instructions. **b** Statistical analysis of the percentage and the absolute cell number of γδ T cells in liver tissue at indicated time points after CCl_4_ treatment. **c**–**e** Whole spleen cell suspensions from C57BL/6 WT mice were stimulated with or without IL-1β (10 ng/mL) for 30 min, **c** representative histograms and **d** MFI of p-S6 (ser235/236) and p-AKT (pS473) in γδ T, Vγ1 and Vγ4 cell subsets in IL-1β treated splenocytes. **e** The MFI was determined by flow cytometry showing the expression of CCR2, CCR4, CCR5, CCR6, CXCR3, CXCR5 and CXCR6 on γδ T cells in IL-1β treated splenocytes. Data are shown as mean ± SD. *P < 0.05, **P < 0.01, and ***P < 0.001 in comparison with the corresponding controls, by unpaired Student’s *t*-test between two groups.

Previous studies indicated that IL-1β is a proinflammatory cytokine that can induce mTOR activation in dermal γδ T cells [20]. We then investigated whether IL-1β also affects mTOR activity in peripheral γδ T cells during CCl_4_-induced liver inflammation. We found that serum IL-1β (Fig. 4a), IL-6 and IL-12 p70 (Supplementary Fig. S8a, b) increased after CCl_4_ treatment, and the kinetics of IL-1β is similar to those of the percentage and absolute numbers of hepatic γδ T cells (Fig. 4b). IL-1β-treated Vγ1 and Vγ4 cells exhibited increased mTORC1 and mTORC2 activity as determined by MFI values for p-S6(ser235/236) and p-AKT(pS473) analysis (Fig. 4c, d), with upregulation of CCR6, CXCR3, CXCR5 and CXCR6 expression (Fig. 4e), but IL-6 and IL-12 have no effects on γδ T cells mTORC2 activation and mobility (Supplementary Fig. S9a, b). These results suggested IL-1β is an important activating factor of mTORC2 in γδ T cells.

### Macrophage-derived IL-1β is essential for γδ T cells accumulation in fibrotic liver

**Fig. 5:**
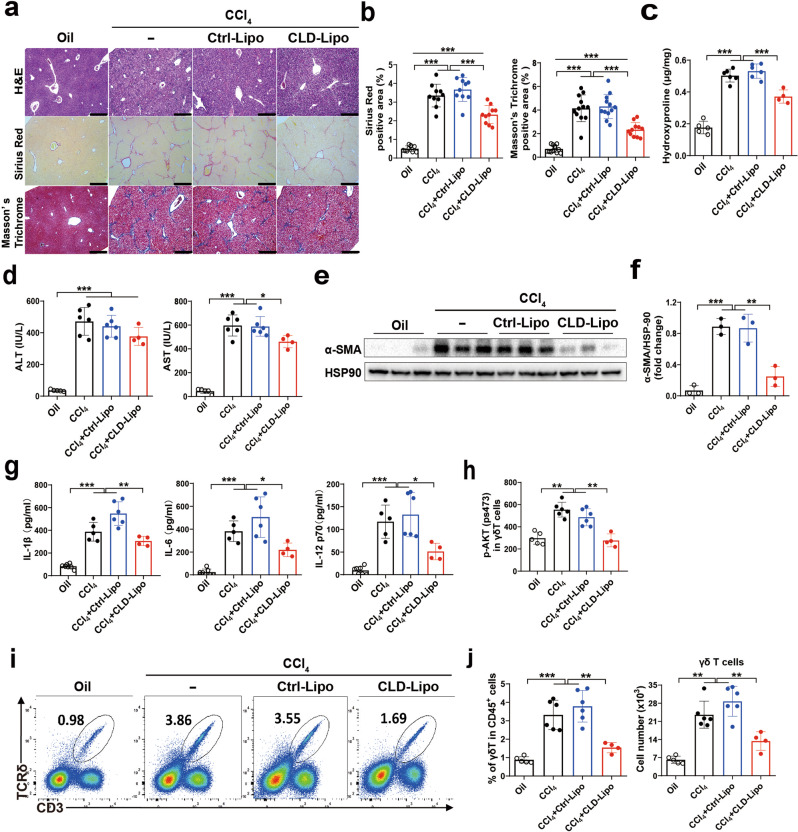
Macrophage-derived IL-1β contributes to γδ T cell migration by activating mTORC2 signaling. **a**–**j** WT mice were administered an intravenous injection of clodronate-loaded liposome (Clo-lip) of 50 mg kg^−1^ 24 h before CCl_4_ treatment; liposome vehicle (lip) served as a control (*n* = 4–6/group; 3 replicates). **a** Representative liver histology of H&E, Sirius Red staining and Masson’s Trichrome staining (bar = 500 μm). **b** Sirius Red staining and Masson’s Trichrome staining were quantified by ImageJ. **c** Hydroxyproline content in liver tissues. **d** Serum ALT and AST levels. **e**, **f** Representative western bolt images and quantitative analysis of α-SMA expression in liver tissues. **g** Serum levels of IL-1β, IL-6 and IL-12 p70 were determined by using ELISA kits. **h** MFI of p-S6 (ser235/236) and p-AKT (pS473) on γδ T in liver tissue. **i** Representative FACS plots. **j** Statistical analysis of the percentage and the absolute cell number of γδ T cells in the liver. Data are shown as mean ± SD. *P < 0.05, **P < 0.01, and ***P < 0.001 in comparison with the corresponding controls, by one-way ANOVA for comparison of multiple groups.

Since macrophage is an important source of IL-1β in liver fibrosis (Supplementary Fig. S10a, b), we hypothesized that macrophage-derived IL-1β activates mTORC2 in γδ T cells. Therefore, we used clodronate-encapsulated liposome (CLD-Lipo) to deplete macrophages (Supplementary Fig. S11), CLD-Lipo-treated mice significantly ameliorated liver fibrosis, as determined by H&E, Sirius Red and Masson’s Trichrome staining, compared with control liposomes (Ctrl-Lipo) treated mice (Fig. 5a, b). Meanwhile, downregulation of hydroxyproline level and AST activity (Fig. 5c, d), and hepatic protein level of α-SMA were markedly decreased in CLD-Lipo-treated mice (Fig. 5e, f).

As a result, depletion of macrophages significantly decreased serum levels of IL-1β, IL-6 and IL-12 p70, which were accompanied by a decrease in mTORC2 activation in hepatic γδ T cells (Fig. 5g, h), suggesting that hepatic macrophage-derived IL-1β activates the mTORC2 of γδ T cells. Furthermore, we detected accumulation of γδ T cells in the fibrotic liver upon CLD-Lipo treatment, the results showed that the proportion and the absolute number of hepatic γδ T cells were decreased at least 2-fold in CLD-Lipo treated mice, compared with Ctrl-Lipo group (Fig. 5i, j).

To further validate hepatic macrophage-derived IL-1β is responsible for γδ T cell migration into liver fibrosis. CCR2^-/-^ mice were used to determine the role of infiltrating macrophages in the fibrotic liver. The results showed that ameliorated fibrosis development was observed in CCR2^-/-^ mice (Supplementary Fig. S12a–f), which were accompanied by decreased the percentage and absolute number of γδ T cells and infiltrating macrophages in the fibrotic liver, compared to WT mice (Supplementary Fig. S12g). As expected, in CCR2^-/-^ mice, the serum levels of IL-1β, IL-6 and IL-12 p70 were decreased after CCl_4_ treatment (Supplementary Fig. S12h). These results suggest that infiltrating macrophage produced IL-1β is important for the induction of mTORC2 activation in γδ T cells, which is required for the accumulation of γδ T cells.

### IFN-γ production by γδ T cells attenuated liver fibrosis via suppression of Th17 cells

**Fig. 6:**
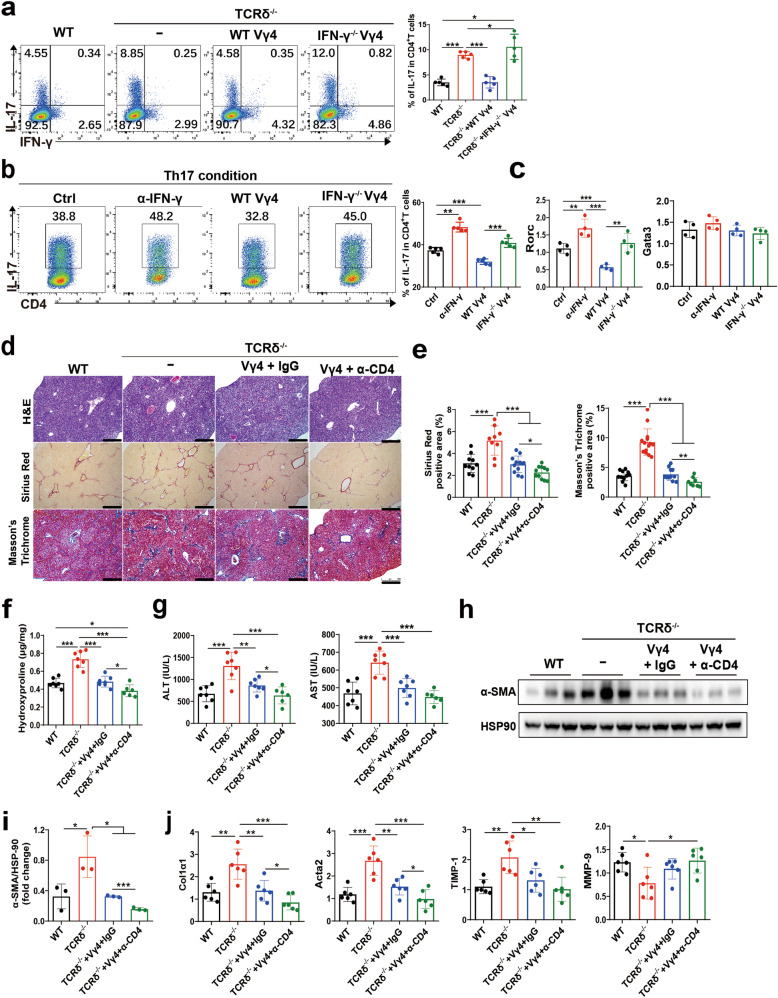
γδ T cells attenuate liver fibrosis via suppression of Th17 cells. **a** Representative FACS plots of IFN-γ and IL-17A in CD4^+^ T cells of WT and TCR δ^-/-^ mice reconstituted with Vγ4 cells from WT or IFN-γ^-/-^ mice after repetitive CCl_4_ challenge for 4 weeks analyzed by flow cytometry (*n* = 5/group; 3 replicates). Lymphocytes were gated on the basis of FAC-A and SSC-A, doublets (FSC-H and FSC-A gating) were excluded from the analysis, and then CD45^+^ CD3e^+^ CD4^+^ CD8^-^FVD^-^ cells were gated and analyzed. **b**, **c** Splenic CD4^+^ T cells from WT mice were cultured in Th17 conditions (anti-CD3 mAb, anti-CD28 mAb, TGF-β, IL-6 and anti-IL-4 mAb) with/without anti-IFN-γ mAb, WT γδ T cells or IFN-γ^-/-^ Vγ4 cells for 96 h. Cells were stained for CD3e, CD4, IL-17A and analyzed by flow cytometry. **b** Representative FACS plots of IL-17A in CD4^+^ T cells, and **c** qRT-PCR analysis of the relative expression of Rorc and Gata3 in CD4^+^ T cells. **d**–**j** WT, TCR δ^-/-^ mice, TCR δ^-/-^ mice reconstituted with Vγ4 cells with/without anti-CD4 treatment after repetitive CCl_4_ challenge for 4 weeks (*n* = 6–7/group; 3 replicates). **d** Representative liver histology of H&E, Sirius Red staining and Masson’s Trichrome staining in liver tissue. **e** Sirius Red staining and Masson’s Trichrome staining were quantified by ImageJ analysis. **f** Hydroxyproline content in liver tissues. **g** Serum ALT and AST levels were measured. **h**, **i** Representative western bolt images and quantitative analysis of α-SMA expression in liver tissues. **j** qRT-PCR analysis of the relative expression of Col1α1, Acta2, TIMP-1 and MMP-9 in liver tissue. Data are representative of at least three independent experiments. Data are shown as mean ± SD. *P < 0.05, **P < 0.01, and ***P < 0.001 in comparison with the corresponding controls, by one-way ANOVA for comparison of multiple groups.

Whether γδ T cells are involved in the regulation of Th17 cells in fibrosis is unknown. We found that Th17 cell frequency is significantly elevated in TCRδ^-/-^ mice in the fibrotic liver, and reconstituted TCRδ^-/-^ mice with WT γδ T cells were able to reduce Th17 levels to those of WT mice (Fig. 6a). Consistently, WT Vγ4 cells inhibited Th17 cell induction in vitro, compared with IFN-γ^-/-^ Vγ4 cells (Fig. 6b). Furthermore, lower mRNA level of Rorc was detected in the coculture of WT Vγ4 cells with CD4^+^T cells compared with that in IFN-γ^-/-^ Vγ4 cells cocultured CD4^+^T cells under Th17 condition, but there was no significant difference in GATA3 mRNA level (Fig. 6c), which indicated that γδ T cells inhibited Th17 cell induction in vitro through IFN-γ production, assuming a direct regulation between IFN-γ^+^ γδ T and Th17 cells. To further verify the interplay between γδ T and CD4^+^ T cells in vivo, we treated γδ T reconstituted TCRδ^-/-^ mice with α-CD4 depleting antibody, the results showed that α-CD4 treatment prevented the development of liver fibrosis compared to the control group (Fig. 6d–j), corroborating that IL-17-producing CD4^+^ T cells play a critical role in the pathogenesis of liver fibrosis, and Th17 differentiation was suppressed by IFN-γ producing Vγ4 cells.

**Fig. 7:**
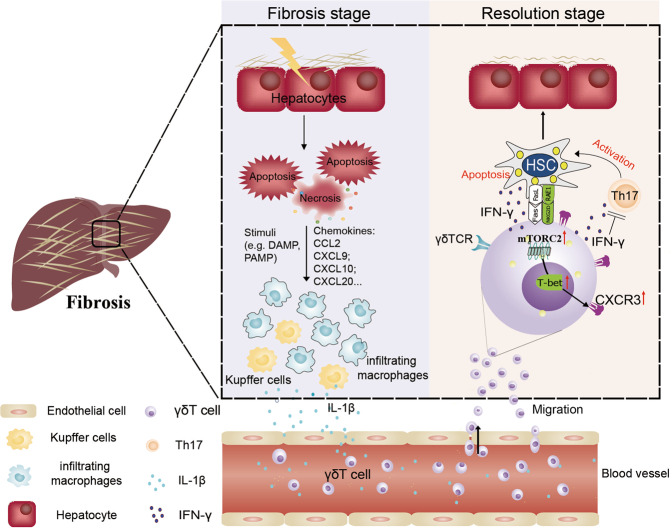
Schematic diagram of mTORC2 signaling mediates γδ T cells migration into the fibrotic liver. The hepatic immune microenvironment mediates chronic damage (e.g., alcohol and virus infection, etc.) induced hepatocyte injury, driving fibrogenesis by HSC activation. Resident hepatic macrophages, Kupffer cells (KC), are an important sensor of tissue injury. They become activated via pathogen-associated molecular patterns (PAMPs) from invading pathogens, by danger-associated molecular patterns (DAMPs) released from injured hepatocytes, proinflammatory cytokine (IL-1β) released from activated KC and infiltrating macrophage (IM) can initiate the γδ T cells CXCR3 expression by inducing mTORC2 activation, which in combination with the chemokines (CXCL9, CXCL10 and CXCL20, etc.) secreted by apoptotic hepatocytes and hepatic macrophages can stimulate the migration of γδ T cells into the fibrotic liver. Moreover, infiltrated γδ T cells exhibited potent cytotoxicity against activated aHSCs by Fas-FasL-dependent manner. Moreover, IFN-γ^+^ γδ T cells producing high levels of IFN-γ suppressed Th17 cell differentiation to ameliorate liver fibrosis. HSC hepatic stellate cells, KC Kupffer cell, IM infiltrating macrophage.

Collectively, we found that mTORC2 signaling, which can be activated by macrophage-derived IL-1β in serum, potentiates CXCR3 expression in Vγ4 cells after CCl_4_ treatment. The upregulation of CXCR3 by T-bet promotes the migration of IFN-γ^+^ γδ T cells into the fibrotic liver, which plays a major protective role by secreting IFN-γ to suppress Th17 differentiation and expressing cytolytic effector molecules (FasL) to exert cytotoxicity against activated HSCs, and ultimately protects the liver from fibrosis (Fig. 7).

## Discussion

Liver fibrosis/cirrhosis is characterized by a complex inflammatory response with tightly regulated interactions between damaged hepatocytes and recruited immune cells [21, 22]. IFN-γ has a protective effect in both mouse and human liver fibrosis by direct inhibition of HSC activation [23, 24], but the initial source of IFN-γ has not been fully elucidated. In this study, we demonstrated that Vγ4 γδ T cells derived IFN-γ play an effective role in ameliorating liver fibrosis by suppressing pro-fibrotic Th17 cells.

Interestingly, naturally occurring IFNγ-producing Th1 cells are almost exclusively CXCR3 positive [25]. In addition, in vitro Th1-polarized T cells highly upregulate CXCR3 [26] and enhanced fibrosis in CXCR3^-/-^ mice was associated with reduced IFN-γ expression and a reduction of IFN-γ–positive intrahepatic T cells [27]. Hence, the tight correlation between CXCR3 expression and Th1 differentiation led us to hypothesize that CXCR3-mediated IFN-γ^+^ γδ T cells recruited to the injured liver; subsequently, our results demonstrated that CXCR3 and its ligands regulate the migration of IFN-γ^+^ γδ T cells. These data suggested that CXCR3 contributed to an increased number of IFN-γ^+^ γδ T cells that were observed during liver fibrosis.

mTOR endows T cells with the ability to properly sense and integrate diverse environmental signals including nutrients and growth factors [28], and was reported to play a pivotal role in diverse immune cells [29–31]. Our previous study found that mTORC1 and mTORC2 have divergent roles in γδ T cell differentiation and mediating the activity of γδ T cells in tumors and autoimmunity [11]. Moreover, a function of mTORC2 in the regulation of migration has been described [32, 33]. To investigate how the mTORC2 signaling in γδ T cells was activated in the fibrotic liver, we focused on IL-1β. One study suggested that IL-1β is a pleiotropic cytokine that induces mTOR-signaling activation in dermal γδ T cells [20]. However, the role of IL-1β in γδ T cells migration remained unclear. In the current study, we demonstrated that IL-1β promoted mTORC2 activation and mobility activity of γδ T cells. Furthermore, we found that the hepatic macrophages were the main source of IL-1β, which is the driving force of mTORC2 activation and migration of γδ T cells into the fibrotic liver. Future detailed cellular and molecular mechanism studies are needed.

Taken together, our study revealed that mTORC2 activation of γδ T cells is dependent on IL-1β producing hepatic infiltrated macrophages, which are important for the migration of IFN-γ^+^ γδ T cells to the fibrotic liver in a CXCR3 dependent manner (Fig. 7). Moreover, we recently reported that transferring allogeneic Vγ9Vδ2 T cells derived from healthy donors into late-stage cancer patients [34], unequivocally validated the clinical safety, and prolong survival in terminal cancer patients. Our work, therefore, highlights the potential therapeutic value of in vitro expanded Vγ9Vδ2 T cells for human liver fibrosis even cirrhosis.

## Supplementary information

Supplementary Information

Checklist

## Data Availability

The published RNAseq data used in this study are available in Sequence Read Archive (SRA) repository at NCBI under the accession number SRP214746. All other data generated or analyzed during this study are included in this published article (and its Supplementary information files). Further inquiries can be directed to the corresponding authors.
