# Supplementary material for: IL-1β-activated mTORC2 promotes accumulation of IFN-γ+ γδ T cells by upregulating CXCR3 to restrict hepatic fibrosis
Source: Cell Death Dis. 2022 Apr 1;13(4):289. doi: 10.1038/s41419-022-04739-3 (PMC8971410; doi:10.1038/s41419-022-04739-3)
Supplement: Supplementary file 1 — Supplementary Information [file 41419_2022_4739_MOESM1_ESM.docx]

**Supporting Information**

**Supplementary Materials and Methods**

**Flow cytometric analysis**

Single-cell suspensions were surface stained in 96-well round plates (Corning) at 2 × 10^6^ lymphocytes per well by incubation with the relevant antibodies diluted in PBS with 2% FBS for 20 minutes at 4℃, followed by 2 washes with FACS buffer (PBS containing 1% bovine serum albumin and 0.04% azide). For intracellular cytokine staining, cells were ﬁrst incubated in RPMI 1640 containing 10% FBS, 50 ng/mL PMA (Sigma-Aldrich), 1ug/ml ionomycin (Sigma-Aldrich) and 0.07% GolgiStop (BD Biosciences) for 4-6 h at 37℃. After the incubation, surface staining was performed as indicated above, cells were incubated in Cytoﬁx/Cytoperm (BD Biosciences) for 20 min, washed in 1 × Permwash (BD Biosciences) and stained with intracellular directly conjugated antibodies for 30 min. For phospho ﬂow staining, p-AKT (pS473) and p-S6 Ribosomal protein (Ser235/236) ﬂow staining, mouse liver non-parenchymal cells were ﬁxed in pre-warmed BD Phosflow Fix Buffer I (BD Biosciences) at 37°C for 10 min, and then washed twice with BD Phosflow Perm/Wash Buffer I (BD Biosciences) and then permeabilized with Perm Buffer III (BD Biosciences) for 60 min on ice. The cells were washed twice with BD Phosflow Perm/Wash Buffer I and stained with fluorochrome-conjugated phospho-Akt (Ser473) or phospho-S6 ribosomal protein (Ser235/236) (Cell Signaling Technology) at 4℃ overnight. FACS was performed with BD FACSVerse^TM^ flow cytometer (San Jose, CA), and data were analyzed with Flowjo 10.0 software.

**H&E**

Liver tissues were fixed with 4 % paraformaldehyde for 24 h, dehydrated, embedded in paraffin and sectioned at 5 μm. The paraffin-embedded sections were stained with hematoxylin and eosin (H&E) according to a standard protocol. In brief, liver paraffin sections (5 μm) were dewaxed as followed: Xylene I for 20 min; Xylene II for 20 min; 100% ethanol I for 5 min; 100% ethanol II for 5 min; 75% ethanol for 5 min; Rinsing with tap water; Stain sections with Hematoxylin solution for 3-5 min, rinse with tap water. Then treat the section with Hematoxylin Differentiation solution, rinse with tap water. Treat the section with Hematoxylin Scott Tap Bluing, rinse with tap water. 85% ethanol for 5 min; 95% ethanol for 5 min; Finally Stain sections with Eosin dye for 5 min. Dehydrate as followed: 100% ethanol I for 5 min; 100% ethanol II for 5 min; 100% ethanol III for 5 min; Xylene I for 5 min; Xylene II for 5 min; Finally seal with neutral gum. Observe with microscope inspection, image acquisition and analysis.

**Sirius Red Staining**

Sirius Red (SR) staining was performed on liver tissue sections to determine liver fibrosis according to a standard protocol. In brief, liver paraffin sections (5 μm) were dewaxed as followed: Xylene I for 20 min; Xylene II for 20 min; 100% ethanol I for 5 min; 100% ethanol II for 5 min; 75% ethanol for 5 min; Rinsing with tap water; Stain the section with Sirius Red solution for 8 min, and then dehydration quickly with two or three cups of anhydrous ethanol. Xylene for 5 min, and seal with neutral gum. Observe with microscope inspection, image acquisition and analysis.

**Serum biochemical analysis**

Blood samples were collected from the orbital sinus. The serum levels of alanine aminotransferase (ALT) and aspartate aminotransferase (AST) were respectively measured using ALT and AST assay kits (Nanjing Jiancheng Bioengineering Institute, Nanjing, China), according to the manufacturer’s protocol.

**Hydroxyproline Assay**

Hydroxyproline from collagen was detected using a hydroxyproline assay kit (ab222941; Abcam) according to the manufacturer’s instructions. Briefly, 50 mg liver tissue was homogenized with 500 ul distilled water, and then added the same volume of 10 N NaOH. The mixture was heated at 120 ℃ for 1 h and then neutralized using 10 N HCl. The precipitant was centrifuged and collected. 33 μg/10 μL samples were then dried on a 65°C hotplate. Measurement at 560 nm absorbance was obtained using a microplate plate reader (Bio-Tek, Winooski, VT, USA), and the following formula was used for calculation: hydrolyzed hydroxyproline concentration = B (amount of hydroxyproline)/V (sample volume) × D (dilution factor).

**Western blot analysis**

 Liver pieces were homogenized in lysis buffer (P0013, Beyotime, Jiangsu, China) supplemented with protease inhibitor cocktail (Roche Diagnostics, IN, USA), and protein concentration was determined using Pierce BCA protein assay kit (Thermo Scientific, MA, USA) according to the manufacturer's instructions. Immunoblotting was performed according to the manufacturer’s guidelines (Bio-Rad, CA, USA). Immunodetection was achieved with chemiluminescence reagent (Thermo Scientific, MA, USA) and detected by a CCD camera-based imager (Bio-Rad, CA, USA). Densitometry analysis was performed using the ImageJ software (National Institutes of Health, MD, USA).

**Chemotaxis assay**

A total of 5 × 10^5^ γδ T cells were suspended in 100 μL of culture medium (RPMI 1640, containing 0.5% BSA and 25 mM Hepes) and placed into the upper chamber of a 24-well transwell plate with a 5-μm-pore filter (Corning Costar). Cells that migrated into the lower chamber, which contained 600 μL of culture medium with or without 30 ng/mL CXCL10 (Peprotech, catalog number: 250-16), were harvested after 3 h and counted. Migration was carried out for 3h at 37 °C. The number of transmigrated γδ T cells were quantified by collecting γδ T cells in both the upper and lower chambers and counting by flow cytometer.

**Cytotoxicity assay**

Vγ1 and Vγ4 γδ T cells were co-cultured with activated HSCs cell line JS1 (1, 2) at an effect (Vγ1 or Vγ4 γδ T cells): target (JS1 cells) (E: T) ratio of 1:2 or 1:5 in 24-well plates at 37 °C in 5% CO_2_. After 4 h incubation, cells were stained with fluorochrome-conjugated CD45 for 20 minutes at 4℃, followed by 2 washes with FACS buffer, propidium iodide (PI; 100 µg/mL) was added for another 15 min to label dead cells, and CD45^-^PI^+^ cells were analyzed by flow cytometry as dead JS1 cells.

**Quantitative Real-time PCR**

Total RNA extraction with TRIzol Reagent (Invitrogen) was performed according to the manufacturer’s instructions and quantified using a NanoDrop 2000c spectrophotometer. RNA was then reverse transcribed to cDNA synthesis using the PrimeScriptTM RT reagent Kit (TaKaRa Dalian Biotechnology Co., Ltd. Dalian, China), and subjected to real-time PCR analysis of gene expression with SYBR Premix Ex Taq (TaKaRa, Dalian, China) with a CFX96 real-time PCR system (Bio-rad, Richmond, CA, USA). Each reaction was performed in triplicate. The results were normalized to GAPDH mRNA expression. The primers used in this study are listed in Table S1.

**Cell preparation, culture and transfer**

γδ T cells were sorted from splenocytes of either WT (wild-type) or Raptor KO or Rictor KO or IFN-γ^-/-^ or IL-17A^-/-^ mice by FACS. Sorted cells were cultured with plate-coated γδ TCR specific Ab (UC7) or Vγ1-specific Ab (2.11) or Vγ4-specific Ab (UC3), respectively (10 μg/mL), and IL-2 (2 ng/mL) for 6 days. The purity of expanded γδ T cells, Vγ1 and Vγ4 γδ T cells were confirmed by FACS analysis. About 2 × 10^5^ expanded γδ T cells or normal saline in the same volume were intravenously injected into TCRδ^−/−^ mice once a week following treatment with CCl4 twice weekly for 4 weeks. For γδ T cells trafﬁcking experiments, 2 × 10^5^ γδ T cells each of in vitro-expanded Ric^f/f^ (CD45.2^+)^ or Ric^KO^ (CD45.2^+)^ were mixed with WT (CD45.1^+)^ γδ T cells in a ratio of 1:1 respectively, and transferred i.v. into TCR δ^-/-^ recipient mice, and then recipient mice were injected (i.p) with a single dose of CCl_4_, γδ T cells inﬁltration of liver was analyzed 48 h after CCl4-administration.

**REFERENCE**

1. Zhang Z, Lin C, Peng L, Ouyang Y, Cao Y, Wang J, Friedman SL, et al. High mobility group box 1 activates Toll like receptor 4 signaling in hepatic stellate cells. Life Sci 2012;91:207-212.

2. Guo J, Loke J, Zheng F, Hong F, Yea S, Fukata M, Tarocchi M, et al. Functional linkage of cirrhosis-predictive single nucleotide polymorphisms of Toll-like receptor 4 to hepatic stellate cell responses. Hepatology 2009;49:960-968.

**Table S1. Primers for Quantitative Real-Time PCR**

| Primer | Sequence (5’— 3’) |
| --- | --- |
| CXCL9 | F: TGAAGTCCGCTGTTCTTTTCC |
| CXCL10  CXCL11  CXCL13  CXCL16  CCL2  CCL5  CCL17  CCL20  CCL22  CCL25  GAPDH  Col1α1  TIPM-1  Acta2  MMP-9 | R: AGTGGATCGTGCCTCGGCTG  F: CAGTGAGAATGAGGGCCATAGG  R: CTCAACACGTGGGCAGGAT  F: GGAAGGTCACAGCCATAGCC  R: GATCTCTGCCATTTTGACGGC  F: GGCCACGGTATTCTGGAAGC  R: GGGCGTAACTTGAATCCGATCTA  F: ACCCTTGTCTCTTGCGTTCTT  R: CAAAGTACCCTGCGGTATCTG  F: TAAAAACCTGGATCGGAACCAAA  R: GCATTAGCTTCAGATTTACGGGT  F: GCTGCTTTGCCTACCTCTCC  R: TCGAGTGACAAACACGACTGC  F: TACCATGAGGTCACTTCAGATGC  R: GCACTCTCGGCCTACATTGG  F: ACTGTTGCCTCTCGTACATACA  R: GAGGAGGTTCACAGCCCTTTT  F: CTCTGCCATCACGTTTAGTGAA  R: GACGGTTATCAAAACAACGCC  F: AGTTCACTGATCCCATAGGCA  R: GCAGGCAAAAAGCCACAGTT  F: GACTTCAACAGCAACTCCCACTC  R: TAGCCGTATTCATTGTCATACCAG  F: CAGGCTGGTGTGATGGGATT  R: AAACCTCTCTCGCCTCTTGC  F: AGAGACACACCAGAGCAGATACCA  R: AGAGACACACCAGAGCAGATACCA  F: ACTGGGACGACATGGAAAAG  R: TTCAGTGGTGCCTCTGTCA  F: GGACCCGAAGCGGACATTG  R: CGTCGTCGAAATGGGCATCT |

**Table S2. Primers in genetic modified mice strains**

| Primer | | Sequence (5’— 3’) |
| --- | --- | --- |
| Raptor flox | F: CTCAGTAGTGGTATGTGCTCAG | |
| Rictor flox  TCRδ^-/-^  hCD2-cre | R: GGG TAC AGT ATG TCA GCA CAG  F: CAAGCATCATGCAGCTCTTC  R: TCCCAGAATTTCCAGGCTTA  Mutant F: CTTGGGTGGAGAGGCTATTC  Mutant R: AGGTGAGATGACAGGAGATC  Wild type F: CAAATGTTGCTTGTCTGGTG  Wild type R: GTCAGTCGAGTGCACAGTTT  Internal Positive Ctrl F: CTAGGCCACAGA ATTGAAAGATCT | |
| CXCR3^-/-^  CCR2^-/-^  Tbx-21^-/-^ | Internal Positive Ctrl R: GTAGGTGGAAATTCTAGCATCATCC  Transgene F: AGATGCCAGGACATCAGGAAC CTG  Transgene R: ATCAGCCACACCAGACACAGA GAT C  Mutant: GGGCCAGCTCATTCCTCCCACTCAT  Wild type: CCACAGGATTTCAGCCTGAACTTTG  Common: CGTGCACTATGCTCAGATATCTGTC  Mutant: CTCGTGCTTTACGGTATCGC  Wild type: GCCCACAAAACCAAAGATGA  Common: CGCAAGGCTATTTGGATTAAGG  Mutant F: GCGCGAAGGGGCCACCAAAGAACGGAG  Wild type: GACTGAAGCCCCGACCCCCACTCCTAAG  Common: TGGGCATACAGGAGGCAGCAACAAATA | |

**Figure S1**

**
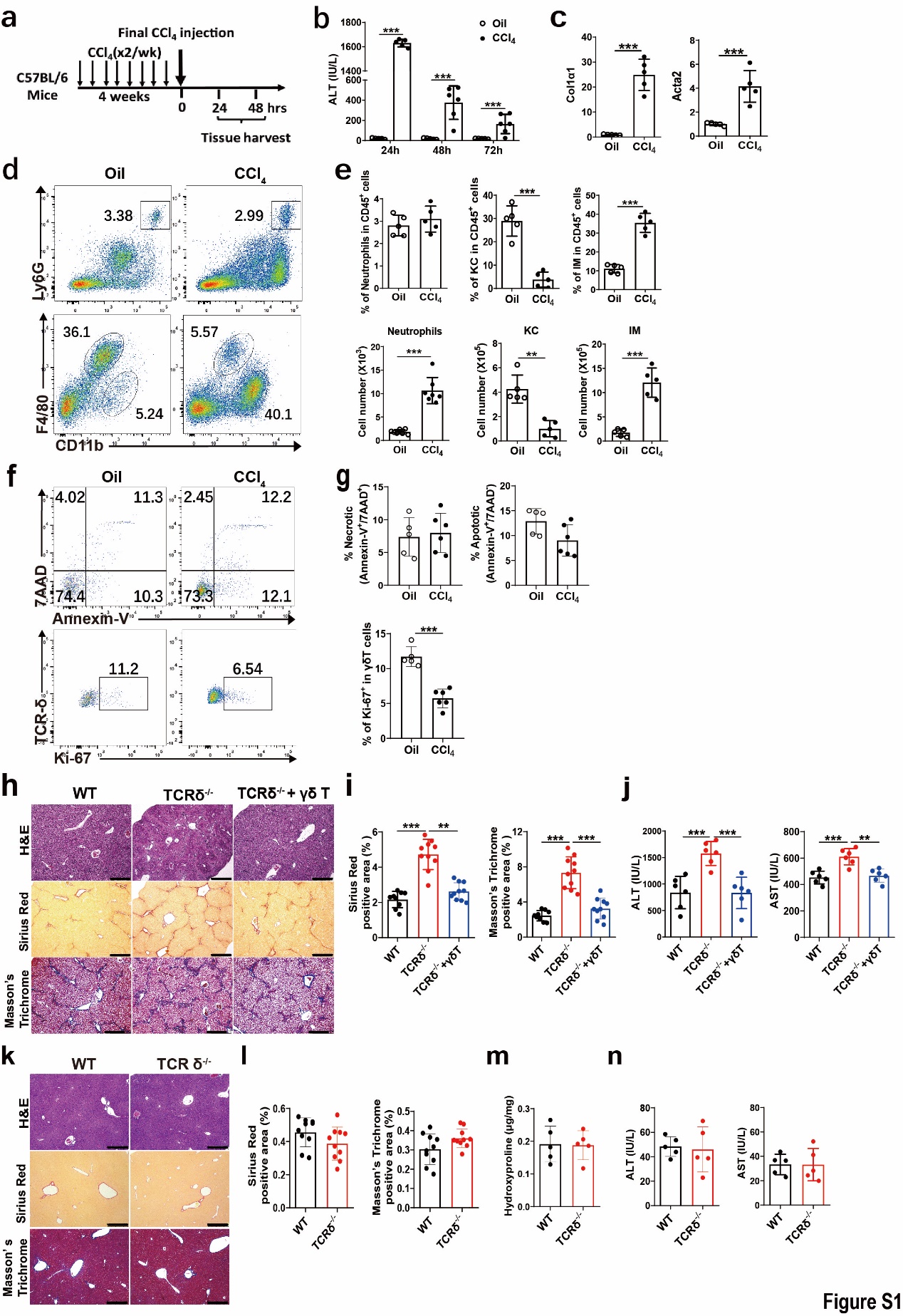
**

**Figure S1. Accumulation of hepatic γδ T cells in chronic liver injury.**

**a** Schematic representation of the model of reversible liver fibrosis in C57BL/6 mice by 4 weeks of twice-weekly i.p. CCl_4_ followed by harvest at 24 or 48 h after the final injection. **b** Serum ALT levels in Oil or CCl4 treated mice and at a stated time point after the final CCl_4_ injection. **c** Whole-liver Acta2 and Col1α1 mRNA levels were measured at 48 h after the final CCl_4_ injection. **d** Representative FACS plots of neutrophils, infiltrating macrophages (IM) and Kupffer cells (KC) in the fibrotic liver after the final Oil or CCl_4_ injection. **e** Quantification of percentage of neutrophils, IM and KC in liver were shown in bar graphs. **f** Representative FACS plots of Annexin-V, 7AAD and Ki-67 staining of hepatic γδ T cells after the final Oil or CCl_4_ injection. **g** Quantification of percentage of apoptotic and proliferating hepatic γδ T cells were shown in bar graphs. **h-j** WT, TCRδ^-/-^ or TCRδ^-/-^ mice reconstituted with 5 × 10^5^ γδ T cells, and repetitive CCl_4_ were challenged twice weekly for 4 weeks (*n* = 5-6/group; 3 replicates). **h** Liver tissues were harvested for H&E, Sirius Red staining and Masson’s Trichrome staining. **i** Sirius Red staining and Masson’s Trichrome staining were quantiﬁed by ImageJ (National Institutes of Health, Bethesda, MD) analysis, counted in 10 different ﬁelds for each sample, 2 samples from each mouse, and presented as fold change compared with the control. **j** Serum levels of ALT and AST were measured at 48 h after the last CCl_4_ injection. **k-n** Normal, untreated WT and TCRδ^-/-^ littermates were used as controls (*n*=5/group; 3 replicates). **k** Livers were harvested for H&E, Sirius Red and Masson’s Trichrome staining. **l** Sirius Red staining and Masson’s Trichrome staining were quantiﬁed by ImageJ. **m** Hydroxyproline content in liver tissues. **n** Serum levels of ALT and AST were measured. Data were presented as the mean ± SD. ***P* < 0.01, and ****P* < 0.001 in comparison with the corresponding controls, by unpaired Student’s t-test between two groups or one-way ANOVA for comparison of two or multiple groups, respectively.

**Figure S2
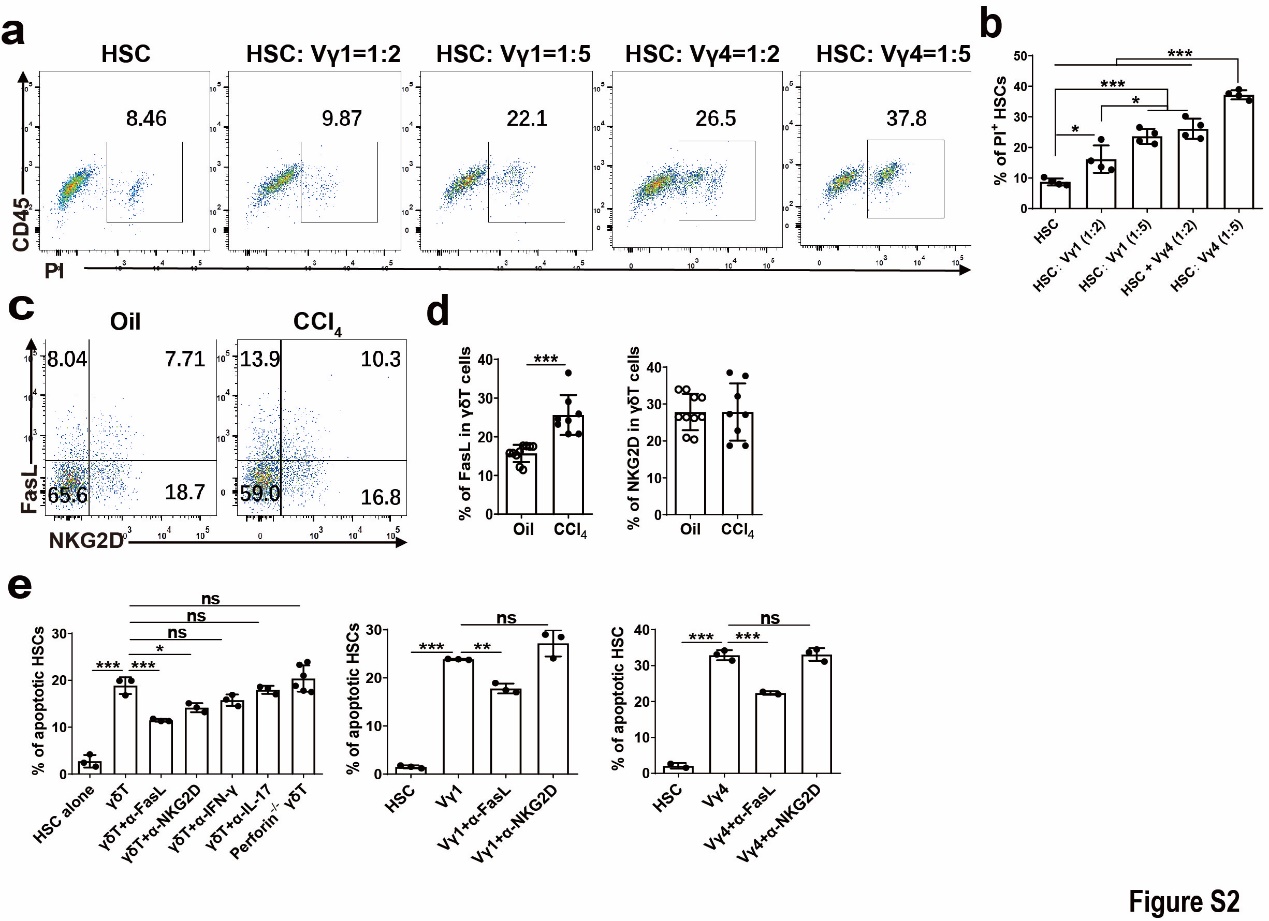
**

**Figure S2 γδ T cells induce apoptosis of activated HSCs.**

**a** Activated HSCs co-cultured with or without γδ T cells for 24 h at different ratio as indicated, representative FACS plots of PI^+^ apoptotic HSCs were analyzed by flow cytometry. **b** Quantiﬁcation of the PI^+^ apoptotic HSCs as shown in a. **c, d** FasL and NKG2D expression on hepatic γδ T cells from WT mice after repetitive Oil or CCl_4_ challenge for 4 weeks (*n* = 8-10/group; 3 replicates). **e** Activated HSCs co-cultured with perforin^-/-^ γδ T cells or WT γδ T cells at an effector: target ratio of 5:1 in the presence or absence of FasL, NKG2D, IFN-γ or IL-17-speciﬁc neutralizing antibodies for 24 h respectively. The cytotoxicity of γδ T cells against activated HSCs was measured using flow cytometer. Data were presented as the mean ± SD. **P* < 0.05, ***P* < 0.01, and ****P* < 0.001 in comparison with the corresponding controls, by unpaired Student’s t-test between two groups or one-way ANOVA for comparison of two or multiple groups, respectively.

**Figure S3**

**
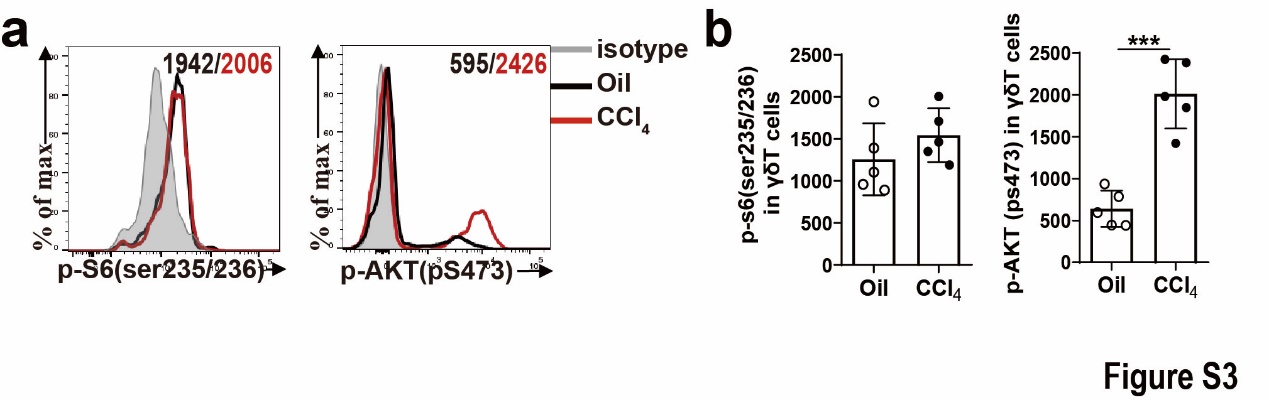
**

**Figure S3.  mTORC2 signaling was activated in peripheral γδ T cells during liver fibrosis.**

C57BL/6 mice were treated with Oil or CCl_4_ twice weekly for 4 weeks (*n*= 5/group; 3 replicates). **a, b** Representative histograms and MFI of p-S6 (ser235/236) and p-AKT (pS473) in γδ T cells in the peripheral blood. Data were shown as mean ± SD. ****P* < 0.001 in comparison with the corresponding controls, by unpaired Student’s t-test between two groups.

**Figure S4**

**
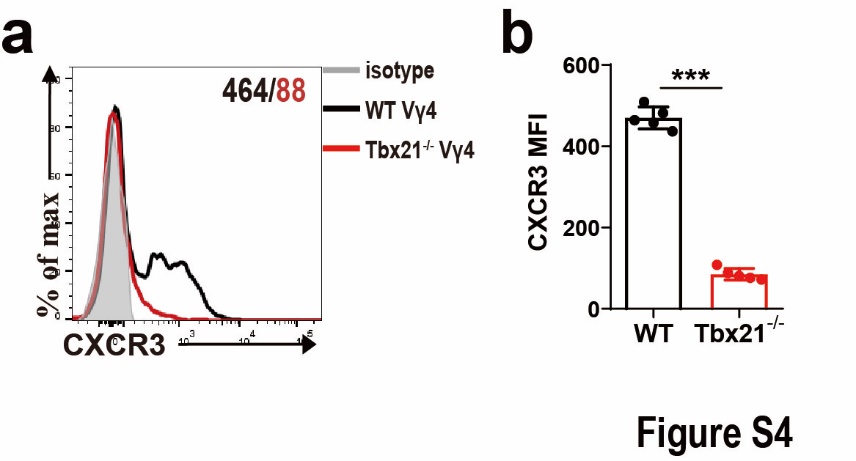
**

**Figure S4.** **The absence of T-bet decreased CXCR3 expression on Vγ4 γδ T cells.**

**a, b** Representative histograms and MFI of CXCR3 on Vγ4 γδ T cells from WT and Tbx21^-/-^ mice. Data were shown as mean ± SD. ****P* < 0.001 in comparison with the corresponding controls, by unpaired Student’s t-test between two groups.

**Figure S5**

**
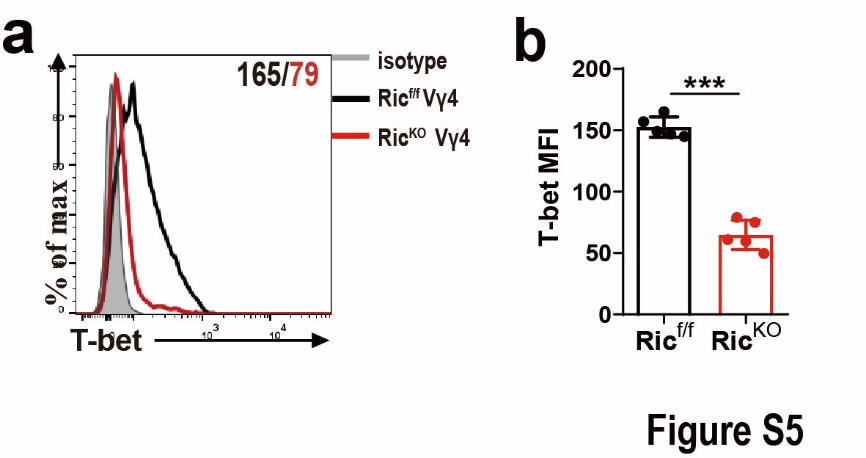
**

**Figure S5. The absence of mTORC2 signaling decreased T-bet expression in Vγ4 γδ T cells**

**a, b** Representative histograms and MFI of T-bet in Vγ4 γδ T cells from Ric^f/f^ and Ric^KO^ mice. Data were shown as mean ± SD. ****P* < 0.001 in comparison with the corresponding controls, by unpaired Student’s t-test between two groups.

**Figure S6**

**
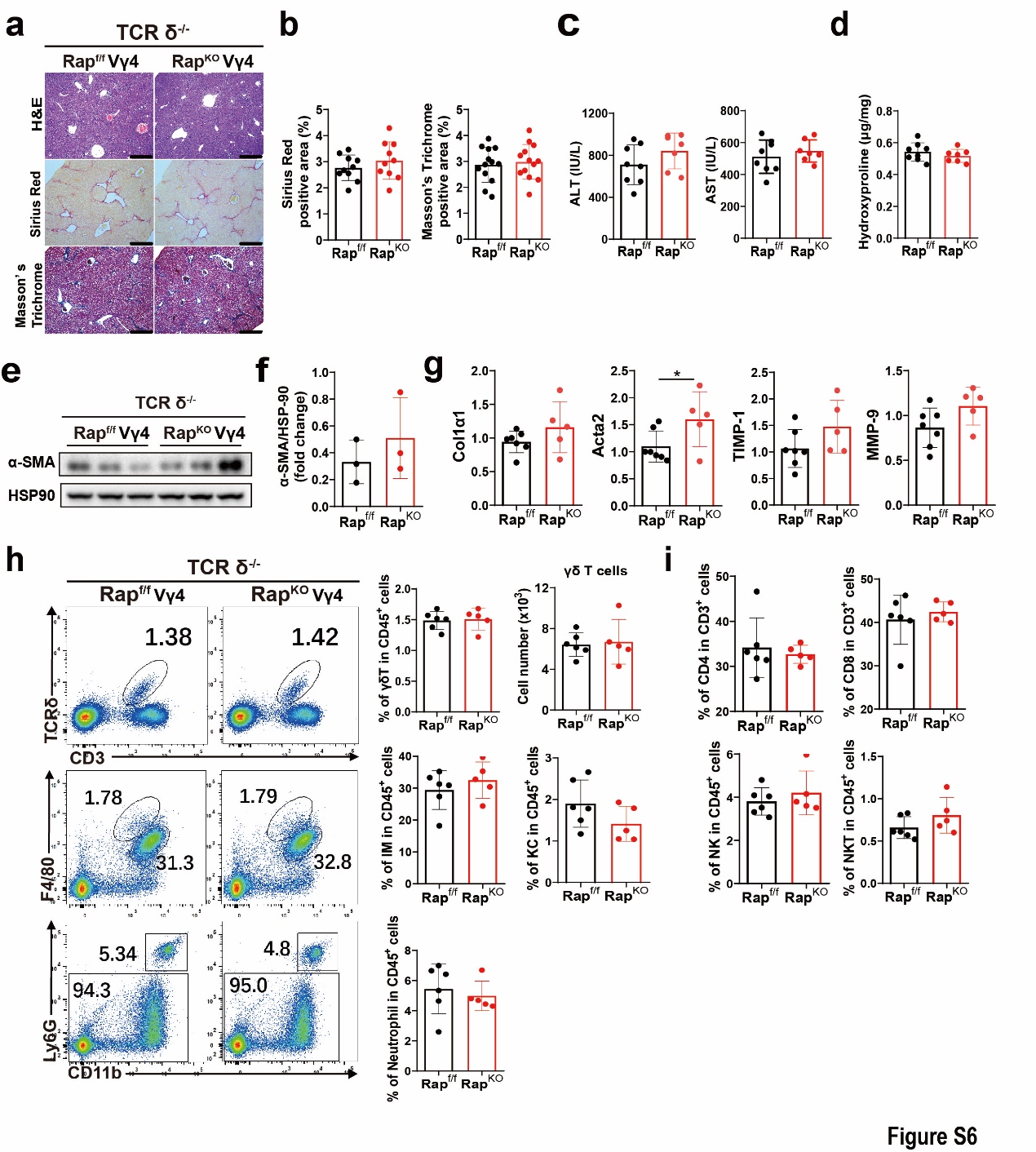
**

**Figure S6. mTORC1 signaling is dispensable for γδ T cells migration.**

TCRδ^-/-^ mice reconstituted with Raptor-f/f Vγ4 or Raptor KO Vγ4 cells, and repetitive CCl_4_ were challenged twice weekly for 4 weeks (*n* = 5-8/group; 3 replicates). **a** Representative liver histology of H&E, Sirius Red staining and Masson’s Trichrome staining. **b** Sirius Red staining and Masson’s Trichrome staining were quantiﬁed by ImageJ. **c** Serum ALT and AST levels. **d** Hydroxyproline content in liver tissues. **e, f** Representative western bolt images and quantitative analysis of α-SMA expression in liver tissues. **g** qRT-PCR analysis of the relative expression of Col1α1, Acta2, TIMP-1 and MMP-9 in mouse liver. **h** Representative FACS plots, statistical analysis of the percentage and the absolute cell number of γδ T cells, neutrophils, KC and IM in the liver. **i** Percentage of CD4^+^ T, CD8^+^ T, NK and NKT cells in the liver. Data were shown as mean ± SD. * *P* < 0.05, ** *P* < 0.01, and *** *P* < 0.001 in comparison with the corresponding controls, by unpaired Student’s t-test between two groups.

**Figure S7**

**
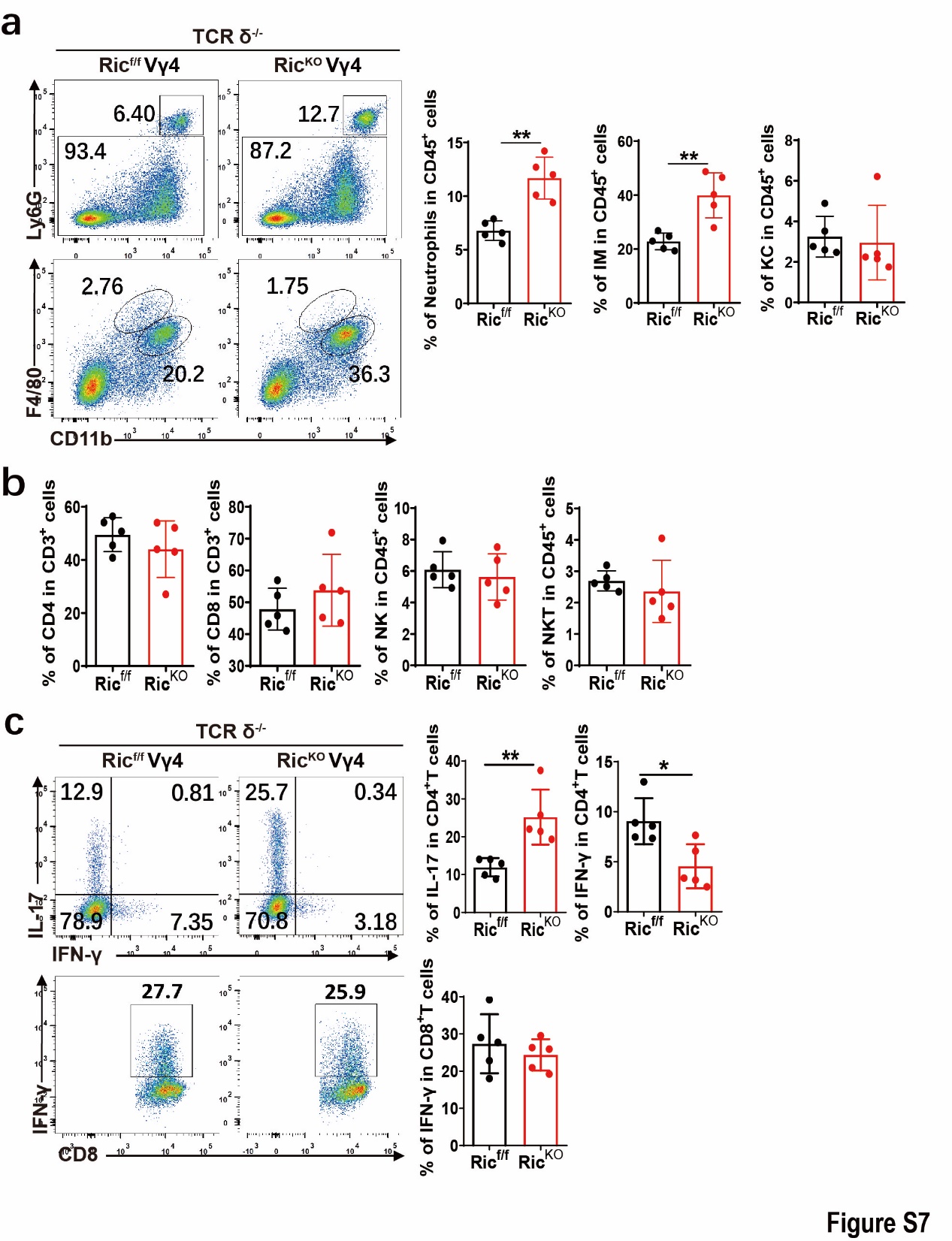
**

**Figure S7 Rictor deficient γδ T cells exacerbated inflammatory cell infiltration in liver fibrosis model.**

TCRδ^-/-^ mice reconstituted with Rictor-f/f Vγ4 or Rictor KO Vγ4 cells, and repetitive CCl_4_ were challenged twice weekly for 4 weeks (*n* = 5/group; 3 replicates). **a** Representative FACS plots and statistical analysis of the percentage of neutrophils, KC and IM in the liver. **b** Percentage of CD4^+^ T, CD8^+^ T, NK and NKT cells in the liver. **c** Representative FACS plots, statistical analysis of the percentage of IFN-γ in CD4^+^ T and CD8^+^ T, IL-17 in CD4^+^ T cells in the liver. Data were shown as mean ± SD. **P* < 0.05 and ** *P* < 0.01 in comparison with the corresponding controls, by unpaired Student’s t-test between two groups.

**Figure S8**

**
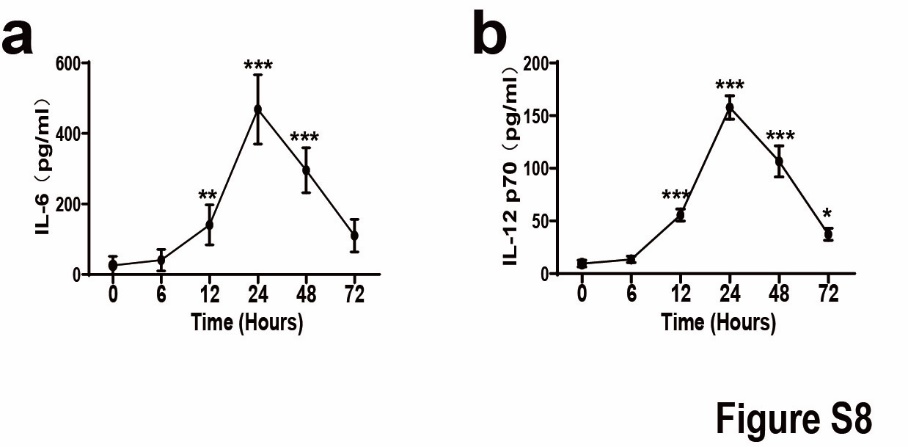
**

**Figure S8. IL-6 and IL-12 p70 were increased after CCl_4_ administration.**

**a, b** Serum was collected at indicated time points after CCl_4_ treatment, and serum levels of IL-6 and IL-12p70 were determined by using ELISA kits. Data were shown as mean ± SD. **P* < 0.05, ***P* < 0.01, and ****P* < 0.001 in comparison with the corresponding controls, by unpaired Student’s t-test between two groups.

**Figure S9**

**
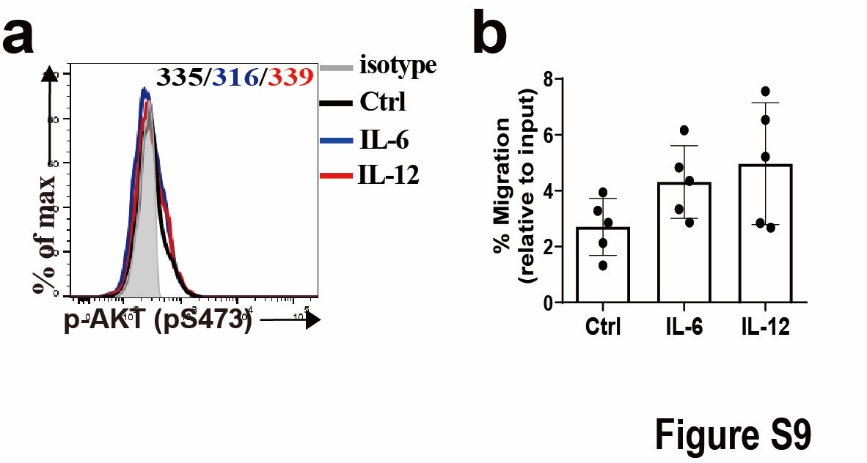
**

**Figure S9. IL-6 or IL-12 p70 did not affect mTORC2 activity and migratory ability of Vγ4 γδ T cells.**

In vitro expanded IFN-γ^+^ Vγ4 cells from IFN-γ-eYPF mice were stimulated with Ctrl PBS, IL-6, or IL-12 respectively. **a** Representative histogram of p-AKT (pS473) in IFN-γ^+^ Vγ4 cells. **b** IFN-γ^+^ Vγ4 cells in response to CXCL10 (100 ng/mL) were assessed in transwell chambers for 3 h.

**Figure S10**

**
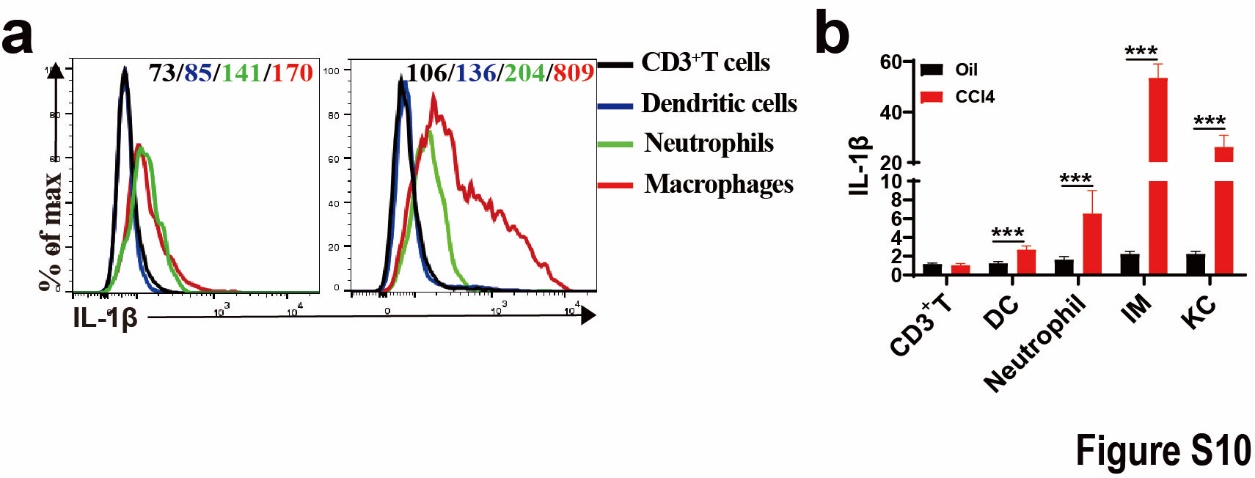
**

**Figure S10. Hepatic macrophages are the major producers of IL-1β after CCl_4_ administration.**

**a** Representative histogram showing IL-1β expression in CD3^+^ T cells, dendritic cells, neutrophils and macrophages at 24 h after the final Oil or CCl_4_ injection. CD3^+^ T cells, dendritic cells, neutrophils, infiltrating macrophage IM and Kupffer cell KC were sorted from Oil or CCl_4_ treated WT mice at 24 h after the final treatment. **b** qRT-PCR analysis of the relative expression of IL-1β in CD3^+^ T cells, dendritic cells, neutrophils, IM and KC. Data were shown as mean ± SD. ****P* < 0.001 in comparison with the corresponding controls, by unpaired Student’s t-test between two groups.

**Figure S11**

**
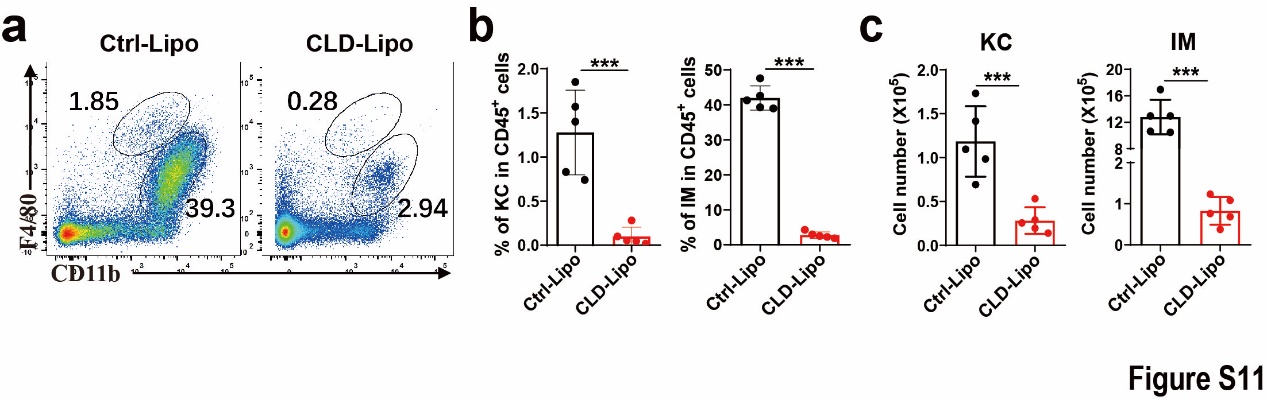
**

**Figure S11 Specific macrophage depletion in the liver by using liposome-encapsulated clodronate.**

**a-c** Representative FACS plots, statistical analysis of the percentage and the absolute number of KC and IM in the liver after intravenous injection of Clodronate-encapsulated liposome CLD-Lipo or control liposomes Ctrl-Lipo (*n* = 5/group; 3 replicates). Data were shown as mean ± SD. *** *P* < 0.001 in comparison with the corresponding controls, by unpaired Student’s t-test between two groups.

**Figure S12**

**
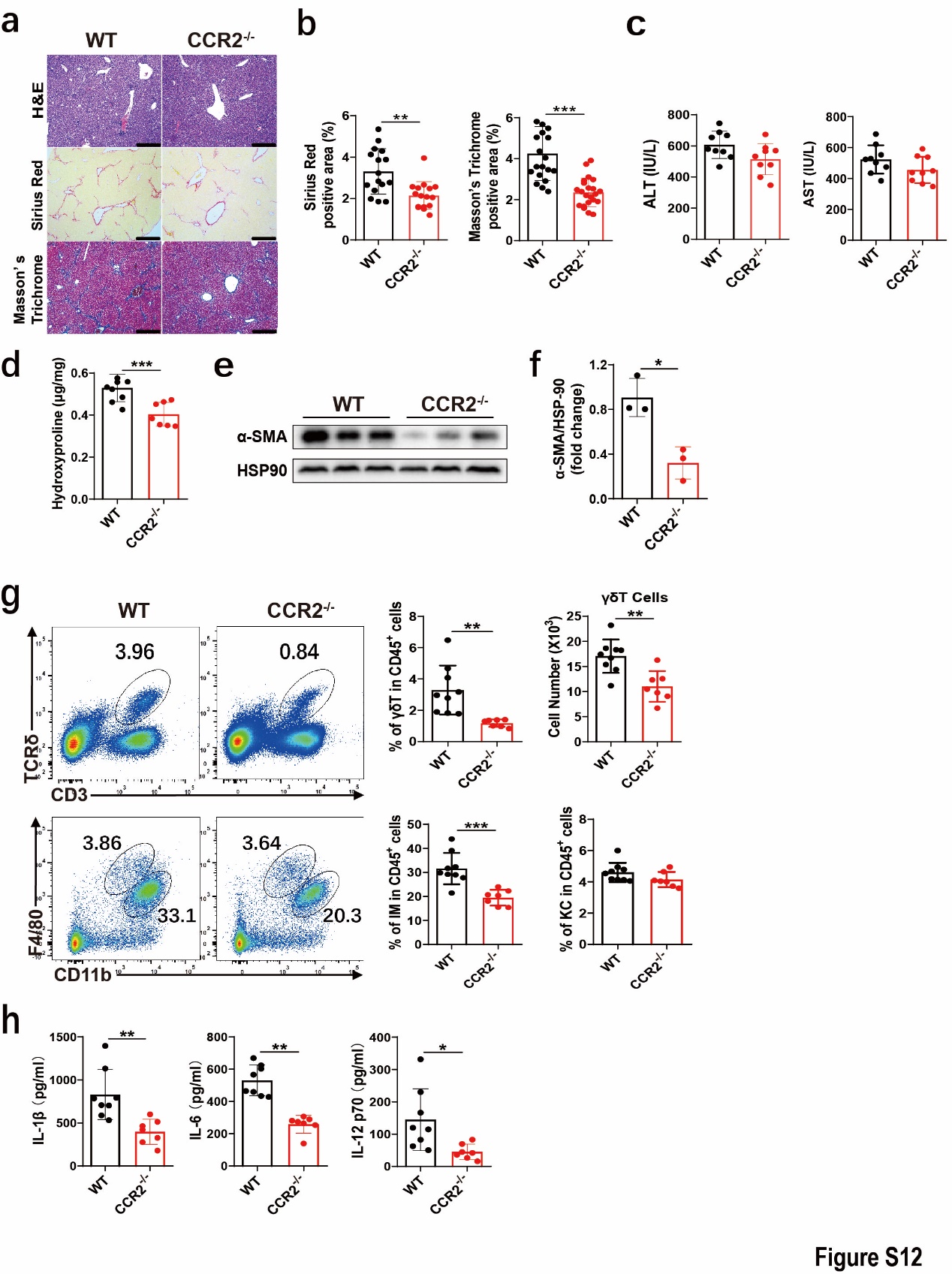
**

**Figure S12 hepatic infiltrating macrophages mediate the accumulation of γδ T cells in fibrotic fibrosis.**

WT and CCR2 KO mice were treated with CCl_4_ twice weekly for 4 weeks (*n* =7-9/group; 3 replicates). **a** Representative liver histology of H&E, Sirius Red staining and Masson’s Trichrome staining. **b** Sirius Red staining and Masson’s Trichrome staining were quantiﬁed by ImageJ. **c** Serum ALT and AST levels. **d** Hydroxyproline content in liver tissues. **e, f** Representative western bolt images and quantitative analysis of α-SMA expression in liver tissues. **g** Representative FACS plots, statistical analysis of the percentage and the absolute cell number of γδ T cells, KC and IM in the liver. **h** Serum was collected at 48 h after the final CCl_4_ injection, and serum levels of IL-1β, IL-6 and IL-12p70 were determined by using ELISA kits. Data were shown as mean ± SD. **P* < 0.05, ** *P* < 0.01, and *** *P* < 0.001 in comparison with the corresponding controls, by unpaired Student’s t-test between two groups.
